# Integrative group psychotherapy reduces daily cortisol output and hair cortisol: A randomized active‑controlled trial with multi‑day profiling

**DOI:** 10.1371/journal.pone.0352095

**Published:** 2026-07-23

**Authors:** Evgeny Pokushalov, Evgeny Terebenin, Andrey Ponomarenko, Yulia Sinyuk, Alexander Samoilov, Tatiana Gigovskaya, Marina Bubnovskaya, Dmitry Kudlay, Elena Trubicina, Claire Garcia, Richard Miller

**Affiliations:** 1 Scientific Research Laboratory, Triangel Scientific, San Francisco, California, United States of America; 2 Center for New Medical Technologies, Novosibirsk, Russia; 3 I.M. Sechenov First Moscow State Medical University (Sechenov University), Moscow, Russia; 4 Novosibirsk State University, Novosibirsk, Russia; Louisiana State University Health Sciences Center Shreveport: LSU Health Shreveport, UNITED STATES OF AMERICA

## Abstract

**Background:**

Chronic psychosocial stress can disrupt hypothalamic-pituitary-adrenal (HPA) axis and sympathetic nervous system function, yet randomized trials rarely combine multi-day diurnal salivary profiling with hair glucocorticoid assessment.

**Methods:**

We conducted a parallel-group, superiority randomized controlled trial with 1:1 allocation to compare an 8-week integrative group program with a dose-matched active control. The intervention combined social-affective skills training, slow breathing with heart-rate-variability biofeedback, and cognitive reappraisal. The primary outcome was the change from baseline (T0) to end of treatment (T1) in daily salivary cortisol output, measured as the area under the curve with respect to ground (AUCg) from 3-day profiles. Key secondary outcomes were the cortisol awakening response, diurnal cortisol slope, salivary cortisone AUCg, salivary alpha-amylase AUCg, and hair cortisol concentration assessed from proximal 1-cm hair segments collected at the end of treatment and at the 6-month follow-up visit, with each segment indexing approximately the month immediately preceding collection. Outcomes were analyzed using intention-to-treat mixed-effects models with false-discovery-rate correction for secondary outcome families.

**Results:**

Sixty participants were randomized to the intervention group (n = 30) or the control group (n = 30). The intervention reduced daily cortisol AUCg more than control from T0 to T1 (between-group difference in change, −21.91 nmol/L·h; 95% CI, −25.87 to −17.95), and this reduction was maintained at T2. Salivary cortisone AUCg and salivary alpha-amylase AUCg also decreased more in the intervention group. Hair cortisol concentration was lower in the proximal 1-cm segments collected at the end of treatment and at the 6-month follow-up visit. Trier Social Stress Test peak cortisol and baseline-corrected AUCi reactivity within the collected −1 to +30 min window did not differ significantly between groups. Adverse events were infrequent and similar between groups.

**Limitations:**

The trial was conducted at a single site, and the Trier Social Stress Test subsample was modest.

**Conclusions:**

An 8-week multicomponent group program reduced daily HPA-axis output and proximal-segment hair cortisol under active-control conditions.

**Trial registration:**

ClinicalTrials.gov NCT06863948 (registered 07 Mar 2025).

## Introduction

Chronic psychosocial stress is linked to dysregulation of the hypothalamic–pituitary–adrenal (HPA) axis and adverse health outcomes [1]. Beyond absolute cortisol levels, dynamic features of the circadian profile are informative: the cortisol awakening response (CAR; the ~ 30–45‑min surge after awakening) and the diurnal decline from morning to bedtime. Meta‑analytic evidence indicates that flatter diurnal slopes are associated with poorer mental and physical health, underscoring the value of high‑quality, repeated ambulatory sampling when testing stress‑reduction interventions [1].

Best‑practice guidance recommends stringent assessment of the CAR—anchored to objective awakening times, with sampling at 0, + 30, and +45 min, adherence checks, and appropriate computation of area‑under‑the‑curve metrics (AUCg, AUCi) [2]. These conventions, together with Pruessner’s trapezoid‑based formulas, have become the field standard for quantifying both total output and change over the early morning window [3]. In parallel, salivary cortisone has emerged as a technically robust complement to salivary cortisol: owing to 11β‑HSD2 activity in salivary glands and higher concentrations at low cortisol levels, cortisone more closely tracks serum free cortisol and improves discrimination when salivary cortisol is near assay limits or contaminated by exogenous hydrocortisone [4].

Whereas salivary measures index state‑level fluctuations across a day, hair cortisol concentration (HCC) provides a retrospective readout of long‑term HPA exposure over weeks to months. Foundational reviews and a large meta‑analysis show that stress‑exposed groups exhibit elevated HCC on average, particularly when stress is ongoing, supporting HCC as a complementary biomarker for chronic load in intervention trials [5,6]. Recent randomized work further indicates that prolonged contemplative mental training can reduce hair glucocorticoids, suggesting that training‑induced changes in allostatic load are detectable at the tissue level [7].

Stress‑reduction interventions, however, have yielded heterogeneous effects on salivary cortisol, likely reflecting variability in targets, dosing, and measurement quality. Early systematic reviews of mindfulness‑based programs were inconclusive for cortisol outcomes, while a more recent meta‑analysis across stress‑management interventions reported overall reductions in cortisol, with larger effects for awakening measures than for diurnal output—implicating both intervention content and design rigor as moderators [8]. Mechanistic specificity also appears critical: in the large‑scale ReSource Project, social‑affective and socio‑cognitive training modules (e.g., compassion, perspective‑taking) attenuated TSST‑evoked cortisol by up to ~50%, whereas attention‑/interoception‑focused practice did not, highlighting the social–evaluative dimension of HPA reactivity [9].

Beyond cognitive and socio‑affective skills, autonomic regulation through slow, resonance‑frequency breathing and heart‑rate‑variability biofeedback (HRVB) targets vagal–baroreflex mechanisms that influence both sympathetic and HPA function. Meta‑analyses and mechanistic reviews indicate that HRVB/slow breathing increase HRV and baroreflex engagement and can reduce stress‑related symptoms; experimental studies show reductions in sympathetic outflow with device‑guided slow breathing. Together, these data justify testing autonomic‑focused components as proximal levers for altering endocrine outcomes [10]. Complementing HPA readouts, salivary α‑amylase (sAA) is a widely used, if nuanced, sympathetic proxy whose interpretation benefits from careful protocol control; incorporating sAA alongside cortisol/cortisone affords a pragmatic two‑axis (HPA/SNS) panel [11].

Methodological considerations are non‑trivial. Adherence lapses around awakening inflate error and can mask intervention effects; objective verification (actigraphy, electronic time‑stamps), multi‑day sampling, and paired cortisone assays mitigate these risks. Likewise, diurnal indices and CAR may be differentially sensitive to intervention content and dosing, arguing for prespecified families of outcomes and robust multiplicity control. Finally, embedding a standardized Trier Social Stress Test (TSST) before and after training allows assessment of peak-latency cortisol reactivity under social evaluation, a key ecological driver of HPA activation [12,13].

Rationale and objective. Building on these insights, we designed a parallel‑group randomized trial to test an integrative group intervention that deliberately combines (i) social‑affective training (empathy/compassion/supportive communication), (ii) autonomic regulation (slow diaphragmatic breathing at ~6 breaths/min with HRV biofeedback), and (iii) cognitive reappraisal/stress‑mindset within mindfulness/interoceptive practice, against a dose‑matched active control devoid of these specific ingredients. We selected a primary physiological endpoint sensitive to daily load—change in salivary cortisol AUCg averaged across three sampling days—alongside key secondaries (CAR AUCi, diurnal slope, salivary cortisone and sAA), hair cortisol concentration from proximal 1-cm segments collected at T0, T1, and the 6-month follow-up visit (T3), with each segment indexing approximately the month before collection, and TSST peak/AUCi cortisol reactivity within the collected sampling window in a substudy. Proximal mechanistic targets (HRV, respiratory rate, cognitive/affective regulation) were prespecified for mediation analyses.

Hypotheses. We hypothesized that, relative to active control, the integrative program would (1) reduce daily cortisol output (AUCg) and HCC, (2) reduce CAR AUCi and alter the wake-to-bed diurnal cortisol slope without assuming that any flattening would be favorable, and (3) dampen TSST peak/AUCi cortisol reactivity within the collected sampling window; and that improvements in autonomic and regulatory target engagement would mediate endocrine changes. By pairing best‑practice CAR/diurnal protocols with a two‑axis salivary panel (cortisol/cortisone + sAA) and hair measures, the trial is positioned to address persistent gaps in the intervention literature—namely, the scarcity of active‑controlled, mechanistically annotated RCTs that integrate multi‑day high‑fidelity sampling with both acute reactivity and chronic‑load biomarkers.

## Materials and methods

### Study design and oversight

We conducted a parallel‑group, randomized, dose‑matched, assessor‑ and laboratory‑blinded clinical trial comparing an integrative group psychotherapy program (intervention, INT) with an active control (CTRL). Assessments occurred at baseline (T0), end of treatment (~8 weeks, T1), post-treatment follow-up (~3 months, T2), and a 6-month follow-up visit (T3). Hair was collected at T0, T1, and T3. At each hair collection, the analyzed proximal 0–1 cm segment indexed approximately the month immediately preceding that collection. The interval between the T1 proximal segment and the proximal segment collected at T3 was not continuously assayed. The full study timeline and assessment schedule are summarized in S1 Fig. The protocol and prespecified statistical analysis plan (SAP) were finalized before enrollment and aligned with CONSORT (including the NPT and Harms extensions), SPIRIT, and TIDieR. The trial was prospectively registered. Recruitment occurred 9 Mar 2025–30 Mar 2025 at the Center for New Medical Technologies, Novosibirsk, Russian Federation.

### Participants

Adults aged 18–60 years with subclinical to moderate anxiety/depression symptoms were eligible. Key exclusions included severe psychopathology, shift work, and major sleep disorders. For women, oral contraceptive status and menstrual cycle phase were recorded and considered at the design/analysis stages. All participants provided written informed consent prior to any procedures. Sixty participants were randomized (INT = 30; CTRL = 30).

### Randomization and masking

Participants were randomized 1:1 using computer‑generated, block‑stratified sequences (strata: sex, baseline symptom severity, and, for women, oral contraceptive status). Allocation was concealed via a centralized assignment system. Outcome assessors and laboratory personnel were blinded to group. Participants were not explicitly informed of allocation; expectancy/masking was evaluated at T1 with a “guess‑the‑group” item and confidence rating.

### Interventions

*Intervention (INT).* The integrative program (8 weekly group sessions, 2–3 h each; 8–12 participants; ≥ 2 therapists) combined three specific components: (1) social‑affective training (empathy, compassion, supportive communication), (2) autonomic regulation (slow diaphragmatic breathing at ~6 breaths/min with HRV biofeedback), and (3) cognitive reappraisal/stress‑mindset, embedded within mindfulness/interoceptive practice. Two blinded coders completed session‑level fidelity/differentiation forms (minutes per component; adherence/competence 0–6; contamination checks). Home practice by component was prescribed and logged weekly.

Treatment delivery followed the preregistered fidelity/differentiation procedures specified in the protocol.

*Active control (CTRL).* A dose‑matched group program emphasized psychoeducation and general stress‑management skills while deliberately omitting the INT‑specific ingredients (no structured compassion dyads, no resonance‑breathing/HRV biofeedback, no targeted reappraisal drills under social‑evaluation). Session content, fidelity coding, and home‑practice tracking paralleled INT procedures.

### Outcomes

*Primary physiological endpoint.* The primary physiological endpoint was the change from T0 to T1 in daily salivary cortisol load, defined as the area under the curve with respect to ground (AUCg, nmol/L·h) averaged across three consecutive sampling days at each wave. Durability of the primary effect was evaluated at T2. AUC metrics were computed using the Pruessner formulas.

*Key secondary physiological endpoints.* Key secondary endpoints were the cortisol awakening response, computed as AUCi from the 0, + 30, and +45 min awakening samples; diurnal cortisol slope (nmol/L per h); salivary α-amylase (sAA) AUCg; and salivary cortisone AUCg. Additional derived diurnal indices, including sAA diurnal slope and selected ratios such as the evening sAA-to-cortisol ratio, were computed as prespecified exploratory measures and are provided in the shared dataset.

*Hair cortisol concentration.* Hair cortisol concentration was assessed from the proximal 0–1 cm segment at T0, T1, and the 6-month follow-up visit (T3). Under the assumption of approximately 1 cm/month hair growth, each segment indexes the month immediately preceding the corresponding collection. HCC at T3 therefore reflects the month before the T3 visit and not the entire T1–T3 follow-up interval.

*Mechanistic targets.* Proximal mechanistic targets assessed at T0 and T1 included resting heart-rate variability indices (RMSSD and HFnu), respiratory rate, ERQ-Reappraisal, Self-Compassion Scale scores, and MAIA-2 interoceptive awareness. A dedicated coping-style or coping-strategy inventory was not administered. Therefore, ERQ-Reappraisal, self-compassion, and MAIA-2 interoceptive awareness were analyzed as target-engagement measures for emotion regulation, self-compassion, and interoceptive awareness; they were not treated as direct measures of coping styles or coping strategies.

*Process, safety, blinding, and assay quality control.* We prospectively collected attendance, coder-rated fidelity/differentiation, home-practice dose, participant guessing of assignment, adverse effects, and assay quality-control metrics. A prespecified Trier Social Stress Test substudy characterized peak and baseline-corrected AUCi cortisol reactivity within the collected −1 to +30 min window before and after the intervention.

### Salivary sampling and preanalytic controls

On each of three consecutive sampling days at each wave, participants collected saliva at awakening (0 min), + 30 min, + 45 min, and bedtime. To improve estimation of daytime exposure while limiting participant burden, an additional mid-day sample (~+6–8 h after awakening) was collected on two of the three days. Target timing windows were: 0 min (±5 min), + 30 and +45 min (±5–10 min), mid-day (±30 min, when scheduled), and evening/bedtime (±30 min). Compliance was supported by actigraphy (7 nights around saliva days) and electronic time‑stamps; preanalytic restrictions included no food/caffeine/alcohol/smoking and no tooth‑brushing per instruction windows. Laboratory QC included duplicates, limits of detection/quantitation (LoD/LoQ), intra‑ and inter‑assay CV%, plate/batch identifiers, freeze–thaw cycles, and time‑to‑freeze documentation. Steroids were quantified in nmol/L and sAA in U/mL.

### Derivation of diurnal endpoints from serial samples

Daily AUCg values for cortisol, cortisone, and salivary α-amylase were computed per day using trapezoidal integration across all available samples collected that day (awakening, + 30, + 45, mid-day when scheduled, and bedtime) and then averaged across the three sampling days to obtain the wave-level endpoint (Pruessner formulas) [3]. The cortisol awakening response was computed as AUCi using the awakening 0/ + 30/ + 45 min samples (expert consensus CAR guidelines) [2] and averaged across days. The diurnal cortisol slope was calculated for each day as (bedtime cortisol − awakening cortisol) divided by the elapsed hours between the corresponding actual sample timestamps. This wake-to-bed definition used only the awakening 0-min and bedtime samples, avoided conflation with the morning CAR rise, and was applied identically to all three sampling days before averaging to obtain the wave-level slope endpoint [14,15].

### Hair cortisol procedures

Hair was collected from the posterior vertex as close as possible to the scalp at T0, T1 (~8 weeks), and the 6-month follow-up visit (T3). We analyzed the proximal 0–1 cm segment (1 cm length). Assuming an average hair growth rate of approximately 1 cm/month, this segment reflects approximately the 4 weeks immediately preceding each collection [5,6]. Under this approximation, the T1 segment reflects approximately weeks 4–8 of the intervention, whereas the T3 segment reflects approximately the month immediately preceding the T3 visit. Accordingly, the “6-month” HCC outcome refers to the timing of sampling at T3 rather than a 6-month retrospective hair segment. The interval between the T1 proximal segment and the proximal segment collected at T3 was not continuously assayed; therefore, HCC at T3 should not be interpreted as an integrated or cumulative measure of the entire follow-up interval. HCC values are reported in pg/mg. We did not collect nail samples; while nail cortisol may capture longer-term cumulative exposure, nail growth dynamics introduce a several-month lag between cortisol incorporation and the clipping available for sampling, and protocols are currently less standardized than for scalp hair cortisol [16].

### Ecological momentary assessment (EMA)

On saliva days, participants received 4–6 prompts/day assessing momentary stress/affect, caffeine/smoking/physical activity, and previous‑night sleep; timestamps enabled alignment with the nearest saliva sample for exploratory within‑day coupling analyses (not part of the primary endpoint). EMA variables relevant to skill‑use and context were prespecified for mechanistic and dose–response models.

### TSST substudy

In a prespecified subset of participants (TSST subsample; n = 30), the Trier Social Stress Test was administered at baseline (pre-intervention) and repeated after the 8-week program (post-intervention) using the standard public-speaking and mental-arithmetic paradigm under social-evaluative threat [13] and the original TSST protocol [12]. Saliva was collected serially for cortisol and α-amylase at t = −20 min (resting baseline), t = −1 min (immediately pre-TSST), and at t = 0, + 10, + 20, and +30 min relative to TSST onset.

To anchor TSST onset to task phases, t = 0 was defined as the start of the evaluative performance period, corresponding to the onset of the speech task. Participants completed a standardized 5-min preparation/anticipation period immediately before t = 0 (t = −5–0), followed by a 5-min speech task (t = 0–5) and a 5-min mental-arithmetic task (t = 5–10). Thus, the active stressor ended at approximately t = +10 min, after which participants remained seated quietly. The t = −1 sample reflects the late-preparation/immediately pre-task baseline; t = +10 corresponds to the immediate post-task time point; and the + 20 and +30 min samples were collected approximately 10 and 20 min after task cessation, respectively.

The sampling points were chosen to capture the expected post-onset salivary cortisol rise and peak-latency window following acute psychosocial stress. The + 20 and +30 min samples therefore characterize peak-latency and early post-task dynamics, not the full HPA-axis recovery curve. No samples were collected at +60 or +90 min; consequently, late recovery and HPA-axis shut-off dynamics were not characterized. This sampling schedule is consistent with TSST methodological guidance and stress-reactivity/recovery reviews emphasizing delayed salivary cortisol peaks and the need to report sampling timing explicitly [17,18].

For each TSST session (pre and post), we report the full time-course trajectory across all collected time points and derive two per-session summary indices within the collected window: peak cortisol, defined as the maximum value across t = 0 to +30 min, and baseline-corrected area under the curve with respect to increase (AUCi), computed by trapezoidal integration over t = −1 to +30 min using the t = −1 min sample as baseline. These indices quantify peak-magnitude and baseline-corrected reactivity within the sampled peak-latency window and do not quantify late recovery.

Although salivary α-amylase was collected during the TSST sessions, the prespecified TSST substudy endpoints reported in this manuscript focus on cortisol (trajectory, peak, and AUCi); the TSST sAA time-series are provided in the shared dataset (S1 Dataset) for transparency.

### Process measures, expectancy/masking, and safety

Session attendance, coder‑rated fidelity/differentiation, and home‑practice minutes were captured prospectively. Expectancy was assessed with CEQ early in treatment; therapeutic alliance/group climate (WAI/GCQ) were collected on schedule. Masking/expectancy was further evaluated by a T1 group‑guess item with confidence. Adverse events and negative effects were monitored with the NEQ and reported per CONSORT‑Harms.

### Sample size and power

The target sample (≈60–80 randomized, 1:1) was based on detectable between‑group differences in change on the primary endpoint, accounting for repeated measures and potential clustering by therapy group/therapist (design effect from assumed intraclass correlation), with allowance for attrition. In this cohort, 60 participants were randomized (INT = 30; CTRL = 30). Detailed power calculations and operating characteristics are provided in the protocol.

### Statistical analysis

The primary intention-to-treat (ITT) analysis followed the prespecified statistical analysis plan (SAP). For diurnal salivary endocrine endpoints, day-level indices were first computed per sampling day (AUCg, CAR AUCi, and diurnal slope as defined in Methods) and then averaged across the three consecutive sampling days to yield one wave-level value per participant per assessment wave (T0 baseline, T1 end of treatment, T2 post-treatment follow-up). Thus, repeated measurements comprised up to three observations per participant for diurnal salivary endpoints.

Primary inference used linear mixed-effects models (LMMs) with fixed effects for time (categorical; T0 as reference), group (INT vs CTRL), and the time×group interaction. The time×group terms estimate between-arm differences in change from baseline at each follow-up wave. Prespecified pre-analytic covariates included awakening time, sampling-window adherence metrics, caffeine/smoking flags, assay plate/batch indicators, freeze–thaw cycles, and, for women, oral-contraceptive status and menstrual cycle phase. Cortisol, cortisone, sAA, CAR AUCi, and HCC outcomes were analyzed on the natural-log scale to address skewness; model coefficients were back-transformed for interpretation as percent ratio-of-change (100×[exp(β)−1]). Diurnal cortisol slope was modeled on the raw scale. These transformations were used for inferential modeling only; descriptive means ± SD, within-group changes, and tabled raw values were retained on the original measurement scale for clinical and physiological interpretability.

To account for within-participant correlation due to repeated measures, models included a participant-level random intercept. To address potential dependency arising from group-based intervention delivery, models additionally included a random intercept for the therapy delivery cluster (therapy group), with therapist effects modeled as nested within therapy group where applicable; random slopes for time were evaluated and retained only if supported by model fit. Models were estimated by (restricted) maximum likelihood; degrees of freedom for fixed effects used Satterthwaite approximations. Given the small number of clusters, we also examined Kenward–Roger degrees of freedom and cluster-robust (sandwich)/ wild-cluster bootstrap standard errors as robustness checks; substantive conclusions were unchanged.

For hair cortisol concentration, the same LMM framework was applied with time levels T0, T1, and T3, where T3 denotes the 6-month follow-up hair collection visit. The modeled HCC value at each time point represents the proximal 0–1 cm segment and not cumulative exposure over the full interval between visits. For TSST outcomes in the substudy, peak cortisol within t = 0 to +30 min was analyzed using a within-participant mixed-effects model including fixed effects for session (post vs pre), group, and session×group, with a participant random intercept. TSST peak cortisol was analyzed on the natural-log scale in the inferential model. Baseline-corrected TSST cortisol AUCi over t = −1 to +30 min was retained as a window-limited summary index and evaluated using the untransformed raw-scale OLS change-score comparison reported in Table 5. Because no samples were collected at +60 or +90 min, these analyses address peak-magnitude and baseline-corrected AUCi reactivity within the sampled window and do not address late recovery or HPA-axis shut-off.

Missing data were handled under ITT using maximum likelihood within the mixed-model framework (i.e., all available observations contributed to estimation under a missing-at-random assumption). Endpoint-level missingness for the prespecified primary and key secondary outcomes was minimal (see CONSORT flow). The mixed-model framework nevertheless accommodates missing observations if present under a missing-at-random assumption. Two-sided α = 0.05 was used for primary hypothesis tests. Multiplicity for prespecified secondary outcome families was controlled using the Benjamini–Hochberg false discovery rate (FDR; q = 0.05).

To evaluate robustness to analytic choice, we additionally performed a conventional mixed-design repeated-measures ANOVA with Time as a within-subject factor and Group as a between-subject factor. This sensitivity analysis was performed on complete cases for the principal repeated physiological outcomes, including diurnal endocrine endpoints, hair cortisol concentration, and autonomic target-engagement indices (RMSSD, HFnu, and resting respiratory rate), using the same transformation rules as in the primary models (S5 Table). For transparency and comparability with prior literature, we also report descriptive between‑group differences in mean change based on the endpoint‑specific individual change scores (Δ) reported in the main outcome tables (e.g., ΔT1–T0, ΔT3–T0, or Δpost–pre, as specified in each table footnote).

All data curation and statistical analyses were performed independently by staff at the Center for New Medical Technologies (Novosibirsk) and by analysts at the Scientific Research Laboratory, Triangel Scientific (San Francisco, USA).

### Tabular reporting strategy

Descriptive means ± SD, within-group changes, and tabled raw values are reported on the original measurement scale for clinical and physiological interpretability. Inferential LMMs used natural-log transformed outcomes where specified, with effects back-transformed for interpretation; raw-scale outcomes, including diurnal slope and non-hormonal target-engagement measures, were modeled on their original scale unless otherwise stated.

For transparency in *Results*, we present: (i) means ± SD at each time point; (ii) within‑group changes as mean (95% CI) using t‑based intervals; and (iii) between‑group differences in mean change (INT–CTRL) with 95% CI and two‑sided *P* values estimated by ordinary least squares on the endpoint‑specific individual change scores (Δ) reported in each table (see table footnotes for the exact Δ definition). These descriptive estimates complement the prespecified mixed‑effects ITT models. Baseline (Table 1), process/fidelity/adherence (S3 Table), and safety/blinding/assay QC (S4 Table) were summarized descriptively; continuous variables were compared using Welch’s t tests and categorical variables using Fisher’s exact test or χ² tests, as appropriate (two-sided).

**Table 1:** Baseline characteristics of the participants at enrollment.

Characteristic	Intervention (n = 30)	Control (n = 30)	Total (n = 60)	P value
Female sex, n (%)	20 (67)	22 (73)	42 (70)	0.779
Age, y (mean ± SD)	32.0 ± 8.0	34.8 ± 7.2	33.4 ± 7.7	0.166
BMI, kg/m² (mean ± SD)	25.8 ± 4.4	27.3 ± 5.3	26.5 ± 4.9	0.246
STAI‑Trait score at baseline (mean ± SD)	46.9 ± 8.8	46.7 ± 8.9	46.8 ± 8.8	0.919
BDI‑II score at baseline (mean ± SD)	24.5 ± 8.3	26.2 ± 7.8	25.4 ± 8.1	0.409

Data are mean ± SD or n (%). P values from Welch’s t test (continuous) or Fisher’s exact test/χ² (categorical), two‑sided. Abbreviations: BDI‑II, Beck Depression Inventory‑II; STAI, State‑Trait Anxiety Inventory.

### Data handling and quality control

Electronic case‑report forms (eCRFs) captured exact awakening times (actigraphy), sample timestamps, “minutes‑from‑wake,” timing‑window flags (≤±5/ ± 10/ ± 30), and reasons for deviations; covariates included menstrual phase/OC status, smoking/caffeine/alcohol/physical activity, and sleep (actigraphy plus diary), as well as laboratory metadata (plate/batch IDs, LoD/LoQ, CV%, freeze–thaw cycles, low‑volume/trace blood flags). Derived variables comprised AUCg/AUCi and diurnal slopes (with multi‑day ICCs as applicable).

### Ethics

The study was conducted according to the guidelines of the Declaration of Helsinki and approved by the Center for New Medical Technologies Ethics Committee (approval number 0182CS_2024, 10 Dec 2024). All participants provided written informed consent prior to any procedures. Adverse events were monitored and adjudicated per CONSORT-Harms.

### Study design

This study was a parallel-group, superiority, randomized, active-controlled trial with a 1:1 allocation ratio.

### Trial registration

The trial was prospectively registered at ClinicalTrials.gov (NCT06863948) on 7 March 2025, and the first participant was enrolled on 9 March 2025.

### Protocol and statistical analysis plan

The trial protocol and prespecified statistical analysis plan are provided as Supporting Information (S1 File).

## Results

### Participants and setting

**Fig 1:**
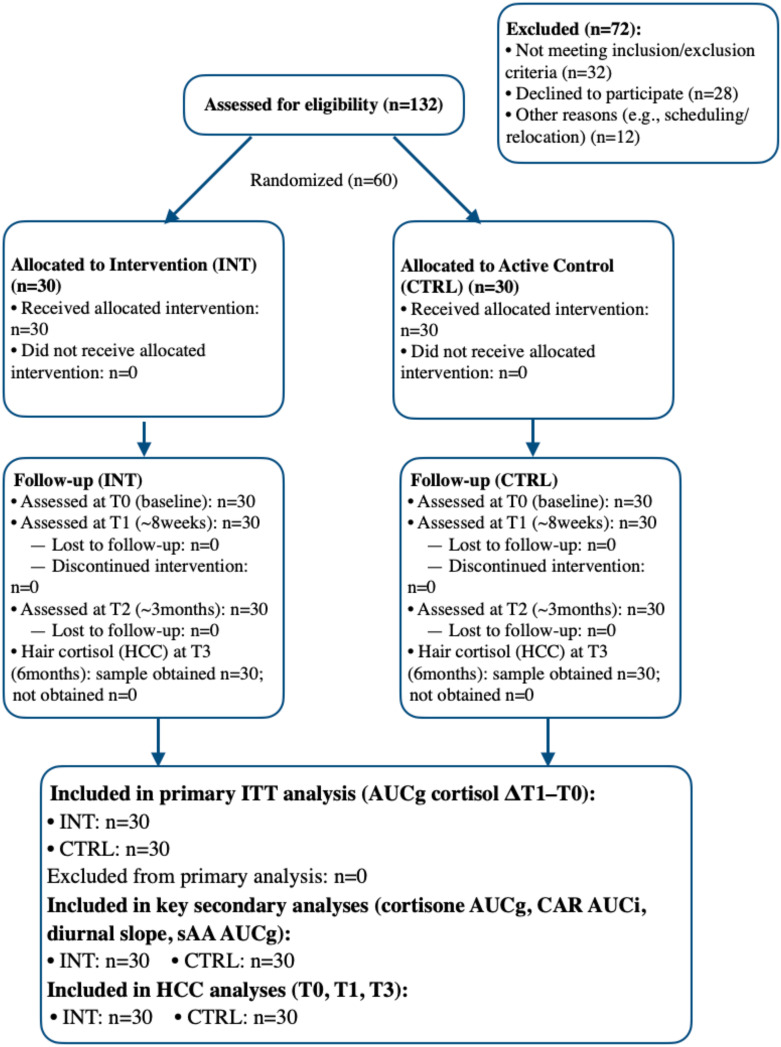
CONSORT participant flow.

A total of 60 participants were randomized (Intervention [INT], n = 30; Control [CTRL], n = 30; Fig 1). Baseline characteristics were well balanced between groups (Table 1). Mean (±SD) age was 33.4 ± 7.7 years, 70% were women, and baseline symptom severity was similar by group (BDI‑II 24.5 ± 8.3 vs 26.2 ± 7.8; STAI‑Trait 46.9 ± 8.8 vs 46.7 ± 8.9; all *P* ≥ 0.16). As prespecified in the protocol, the full analysis focused on change from baseline (T0) to end of treatment (T1) with persistence at T2 and long‑term follow‑up for hair cortisol at T3.

The randomized trial was conducted at the Center for New Medical Technologies (Novosibirsk, Russian Federation), which provided clinical infrastructure for participant recruitment, study visits, sample handling, and on‑site data management in accordance with the prespecified protocol.

### Primary outcome: Daily cortisol load (AUCg)

All diurnal endocrine outcomes represent wave-level endpoints derived from serial saliva sampling across three consecutive days; daily indices were computed per day and then averaged across days as specified in Methods. The prespecified primary endpoint—change in daily salivary cortisol AUCg from T0 to T1—favored INT (Table 2). Mean (±SD) AUCg decreased from 129.35 ± 16.71 to 91.35 ± 13.96 nmol/L·h in INT and from 136.81 ± 20.10 to 120.73 ± 17.73 nmol/L·h in CTRL. The between‑group difference in mean change (INT–CTRL) was −21.91 nmol/L·h (95% CI, −25.87 to −17.95; *P* < 0.001). Reductions were maintained at T2 (INT 86.63 ± 13.06 vs CTRL 117.56 ± 17.34 nmol/L·h).

**Table 2:** Primary and key secondary salivary outcomes.

Outcome	Intervention	Control	Between‑group difference in change (INT–CTRL)
AUCg (salivary cortisol), T0 (nmol/L·h)	129.35 ± 16.71	136.81 ± 20.10	
AUCg (salivary cortisol), T1 (nmol/L·h)	91.35 ± 13.96	120.73 ± 17.73	
AUCg (salivary cortisol), T2 (nmol/L·h)	86.63 ± 13.06	117.56 ± 17.34	
AUCg (salivary cortisol), ΔT1–T0 (nmol/L·h)	−37.99 (−42.07 to −33.92)	−16.08 (−17.93 to −14.22)	−21.91 (−25.87 to −17.95); *P* < 0.001
AUCg (salivary cortisone), T0 (nmol/L·h)	326.46 ± 46.18	336.57 ± 50.27	
AUCg (salivary cortisone), T1 (nmol/L·h)	233.19 ± 31.90	302.82 ± 41.45	
AUCg (salivary cortisone), T2 (nmol/L·h)	220.15 ± 31.48	296.40 ± 40.33	
AUCg (salivary cortisone), ΔT1–T0 (nmol/L·h)	−93.27 (−106.15 to −80.39)	−33.75 (−57.54 to −9.96)	−59.52 (−89.54 to −29.50); *P* < 0.001
CAR AUCi (cortisol), T0 (nmol/L·h)	2.19 ± 0.80	1.96 ± 0.77	
CAR AUCi (cortisol), T1 (nmol/L·h)	1.20 ± 0.73	1.71 ± 0.72	
CAR AUCi (cortisol), T2 (nmol/L·h)	1.10 ± 0.60	1.66 ± 0.67	
CAR AUCi (cortisol), ΔT1–T0 (nmol/L·h)	−0.99 (−1.29 to −0.69)	−0.24 (−0.39 to −0.09)	−0.75 (−1.01 to −0.49); *P* < 0.001
AUCg (salivary α‑amylase), T0 (U/mL·h)	1436.82 ± 355.89	1408.49 ± 339.41	
AUCg (salivary α‑amylase), T1 (U/mL·h)	1121.49 ± 280.07	1251.71 ± 295.39	
AUCg (salivary α‑amylase), T2 (U/mL·h)	1091.05 ± 256.02	1214.93 ± 284.31	
AUCg (salivary α‑amylase), ΔT1–T0 (U/mL·h)	−315.33 (−367.97 to −262.69)	−156.78 (−197.48 to −116.07)	−158.55 (−223.68 to −93.43); *P* < 0.001
Diurnal slope (cortisol), T0 (nmol/L per h)	−0.639 ± 0.212	−0.735 ± 0.170	
Diurnal slope (cortisol), T1 (nmol/L per h)	−0.503 ± 0.157	−0.661 ± 0.141	
Diurnal slope (cortisol), T2 (nmol/L per h)	−0.477 ± 0.161	−0.633 ± 0.159	
Diurnal slope (cortisol), ΔT1–T0 (nmol/L per h)	+0.136 (0.097 to 0.176)	+0.073 (0.043 to 0.103)	+0.063 (0.015 to 0.111); *P* = 0.011

Wave-level values are averages of day-level indices computed across three consecutive sampling days. Daily AUCg values were computed by trapezoidal integration across the serial samples collected that day (awakening, + 30, + 45, mid-day when scheduled, and bedtime). CAR AUCi used awakening 0/ + 30/ + 45 min samples. Diurnal slope used only the awakening 0-min and bedtime samples and was calculated for each day as (bedtime cortisol − awakening cortisol) divided by the elapsed hours between the actual sample timestamps; the three day-level slopes were then averaged to obtain the wave-level endpoint. Descriptive values at each time point are untransformed raw-scale mean ± SD; within-group changes and between-group differences are untransformed raw-scale mean changes with 95% CI. The rightmost column reports the between-group difference in mean change (INT–CTRL) with 95% CI and two-sided P values from ordinary least squares on Δ(T1–T0). These descriptive estimates complement the prespecified mixed-effects models; cortisol, cortisone, sAA, and CAR AUCi were modeled on the natural-log scale, whereas diurnal slope was modeled on the raw scale (S1 Table). Abbreviations: AUCg, area under the curve with respect to ground; AUCi, area under the curve with respect to increase; CAR, cortisol awakening response; sAA, salivary α-amylase. Units as indicated.

### Key secondary salivary outcomes

Results were directionally consistent across complementary salivary markers (Table 2). Salivary cortisone AUCg decreased more in INT than in CTRL, with a between-group difference in change of −59.52 nmol/L·h (95% CI, −89.54 to −29.50; P < 0.001). The cortisol awakening response also decreased more in INT, with a between-group difference in CAR AUCi change of −0.75 nmol/L·h (95% CI, −1.01 to −0.49; P < 0.001). The wake-to-bed diurnal cortisol slope became less negative in both groups. The mean ΔT1–T0 shift was + 0.136 nmol/L per h in INT and +0.073 nmol/L per h in CTRL, yielding a between-group difference in change of +0.063 nmol/L per h (95% CI, 0.015 to 0.111; P = 0.011). Salivary α-amylase AUCg decreased more in INT, with a between-group difference in change of −158.55 U/mL·h (95% CI, −223.68 to −93.43; P < 0.001).

To decompose the slope finding, we examined absolute cortisol concentrations at awakening and bedtime (S6 Table). Bedtime cortisol did not increase in the intervention group; it decreased from 2.49 ± 0.94 nmol/L at T0 to 1.91 ± 0.76 nmol/L at T1 and 1.87 ± 0.59 nmol/L at T2. Awakening cortisol decreased more strongly, from 12.00 ± 3.17 nmol/L at T0 to 9.42 ± 2.38 nmol/L at T1 and 9.03 ± 2.34 nmol/L at T2. In CTRL, awakening cortisol decreased from 13.24 ± 2.62 to 11.97 ± 2.26 and 11.68 ± 2.32 nmol/L across T0, T1, and T2, whereas bedtime cortisol was stable or slightly lower at 2.21 ± 0.82, 2.10 ± 0.90, and 2.19 ± 0.92 nmol/L. Thus, the less-negative slope reflects a smaller morning-to-evening amplitude driven primarily by lower awakening and early-morning cortisol values rather than by elevated bedtime cortisol.

### Intention‑to‑treat mixed‑effects models

As prespecified, primary inference was based on linear mixed-effects models with fixed effects for time (T0, T1, and T2), group, and the time×group interaction; random intercepts for participant and therapy delivery cluster; and pre-analytic covariates, including awakening time, sampling-window compliance, caffeine/smoking flags, assay plate/batch, and freeze-thaw cycles. For model-based inference, hormone outcomes were analyzed on the natural-log scale where prespecified and back-transformed for interpretation. The descriptive means and change estimates in the Results text and main tables are untransformed raw-scale values unless explicitly identified as model coefficients. Model-based results converged with the descriptive differences in change reported in the main tables and Supporting Information (S1 Table and S2 Table). A conventional mixed-design repeated-measures ANOVA sensitivity analysis showed the same pattern of Time×Group effects for the main endocrine outcomes, hair cortisol concentration, and autonomic target-engagement indices (S5 Table).

In the primary daily cortisol AUCg model, the T1 × INT interaction was β = −0.223 (SE 0.045; 95% CI, −0.311 to −0.136; P < 0.001), corresponding to a 20.0% lower ratio of change versus control after back-transformation. The T2 × INT interaction was β = −0.249 (SE 0.049; 95% CI, −0.346 to −0.152; P < 0.001), corresponding to a 22.1% lower ratio of change versus control.

For salivary cortisone AUCg, the T1 × INT interaction was β = −0.232 (SE 0.068; 95% CI, −0.367 to −0.097; P < 0.001), corresponding to a 20.7% lower ratio of change versus control. The T2 × INT interaction was β = −0.269 (SE 0.073; 95% CI, −0.412 to −0.126; P < 0.001), corresponding to a 23.6% lower ratio of change versus control.

For CAR AUCi, the T1 × INT interaction was β = −0.466 (SE 0.118; 95% CI, −0.698 to −0.234; P < 0.001), corresponding to a 37.2% lower ratio of change versus control. The T2 × INT interaction was β = −0.511 (SE 0.131; 95% CI, −0.770 to −0.252; P < 0.001), corresponding to a 40.0% lower ratio of change versus control.

For salivary α-amylase AUCg, the T1 × INT interaction was β = −0.130 (SE 0.045; 95% CI, −0.218 to −0.042; P = 0.004), corresponding to a 12.2% lower ratio of change versus control. The T2 × INT interaction was β = −0.148 (SE 0.049; 95% CI, −0.244 to −0.052; P = 0.002), corresponding to a 13.8% lower ratio of change versus control.

For diurnal cortisol slope, which was modeled on the raw scale after uniform wake-to-bed derivation, the T1 × INT interaction was b = +0.067 nmol/L per h (SE 0.022; 95% CI, 0.024 to 0.110; P = 0.002), and the T2 × INT interaction was b = +0.062 nmol/L per h (SE 0.023; 95% CI, 0.017 to 0.107; P = 0.006). Because flatter slopes have been linked to poorer health outcomes in prior meta-analytic work, this less-negative shift is reported as a descriptive change in profile geometry and is not interpreted as evidence of physiological improvement.

For hair cortisol concentration, the T1 × INT interaction was β = −0.143 (SE 0.041; 95% CI, −0.223 to −0.064; P < 0.001), corresponding to a 13.3% lower ratio of change versus control. The T3 × INT interaction was β = −0.201 (SE 0.052; 95% CI, −0.303 to −0.099; P < 0.001), corresponding to an 18.2% lower ratio of change versus control.

In the Trier Social Stress Test substudy, the post×INT interaction for peak cortisol was β = −0.041 (SE 0.030; 95% CI, −0.100 to 0.018; P = 0.17), indicating no statistically significant between-group difference in peak cortisol change within the collected window. Baseline-corrected TSST cortisol AUCi over t = −1 to +30 min was evaluated using the untransformed raw-scale OLS change-score comparison reported in Table 5; this comparison also showed no statistically significant between-group difference. These analyses address peak/AUCi reactivity within the sampled window and do not characterize late recovery. Across models, cluster effects were modest (ICC ≈ 0.02–0.04), and conclusions were unchanged when Satterthwaite degrees of freedom and wild-cluster bootstrap standard errors were used. False-discovery-rate control at q = 0.05 was applied to prespecified secondary outcome families.

### Hair cortisol concentration in proximal 1-cm segments

Because each HCC value was derived from the proximal 0–1 cm hair segment, it reflects approximately the month immediately preceding the respective hair collection. The interval between the T1 proximal segment and the proximal segment collected at T3 was not continuously assayed. HCC declined in both arms, with a greater reduction in INT at both the T1 and T3 collection points (Table 3). For ΔT1–T0, the between-group difference in change was −1.54 pg/mg (95% CI, −1.88 to −1.21; P < 0.001). For ΔT3–T0, where T3 denotes the 6-month follow-up collection visit rather than a 6-month retrospective hair segment, the between-group difference in change was −2.09 pg/mg (95% CI, −2.60 to −1.59; P < 0.001).

**Table 3:** Hair cortisol outcomes.

Outcome	Intervention	Control	Between‑group difference in change (INT–CTRL)
Hair cortisol, T0 (pg/mg)	11.38 ± 4.51	9.11 ± 3.54	
Hair cortisol, T1 (pg/mg)	9.41 ± 3.85	8.68 ± 3.39	
Hair cortisol, T3 (pg/mg)	8.51 ± 3.43	8.33 ± 3.28	
ΔT1–T0 (pg/mg)	−1.97 (−2.29 to −1.65)	−0.43 (−0.55 to −0.31)	−1.54 (−1.88 to −1.21); *P* < 0.001
ΔT3–T0 (pg/mg)	−2.87 (−3.35 to −2.40)	−0.78 (−0.98 to −0.58)	−2.09 (−2.60 to −1.59); *P* < 0.001

HCC was measured from the proximal 0–1 cm hair segment at each time point, approximating the ~ 1 month immediately preceding each collection under the assumption of ~1 cm/month hair growth. T3 denotes the 6-month follow-up hair collection visit, not a 6-month retrospective hair segment. The interval between the T1 proximal segment and the proximal segment collected at T3 was not continuously assayed. Descriptive values at each time point are untransformed raw-scale mean ± SD in pg/mg; within-group changes and between-group differences are untransformed raw-scale mean changes with 95% CI. The rightmost column reports the between-group difference in mean change (INT–CTRL) with 95% CI and two-sided P values from ordinary least squares on individual change scores (ΔT1–T0 and ΔT3–T0). Inferential LMMs for HCC used natural-log transformed HCC and back-transformed ratios of change (S2 Table).

### Mechanistic target engagement

Prespecified proximal targets of the intervention improved in the expected directions from T0 to T1 (Table 4). Resting RMSSD increased more in the intervention group than in the control group, with a between-group difference in change of +5.52 ms (95% CI, 3.80 to 7.23; P < 0.001). Resting HFnu also increased more in the intervention group, with a between-group difference in change of +7.81 normalized units (95% CI, 5.79 to 9.82; P < 0.001). ERQ-Reappraisal improved more in the intervention group, with a between-group difference in change of +0.48 points (95% CI, 0.27 to 0.68; P < 0.001). Self-compassion and interoceptive awareness also increased more in the intervention group, with between-group differences in change of +0.32 points for the Self-Compassion Scale (95% CI, 0.21 to 0.43; P < 0.001) and +0.29 points for MAIA-2 (95% CI, 0.17 to 0.42; P < 0.001).

**Table 4:** Target engagement (Mechanistic) outcomes (T0 to T1).

Outcome	Intervention	Control	Between‑group difference in change (INT–CTRL)
RMSSD rest, T0 (ms)	31.78 ± 8.93	32.31 ± 7.92	
RMSSD rest, T1 (ms)	39.34 ± 8.83	34.35 ± 7.79	
RMSSD rest, ΔT1–T0 (ms)	+7.56 (6.15 to 8.96)	+2.04 (1.00 to 3.08)	+5.52 (3.80 to 7.23); *P* < 0.001
HFnu rest, T0	44.24 ± 17.15	50.18 ± 12.83	
HFnu rest, T1	52.39 ± 18.13	50.52 ± 12.85	
HFnu rest, ΔT1–T0	+8.15 (6.48 to 9.81)	+0.34 (−0.87 to 1.55)	+7.81 (5.79 to 9.82); *P* < 0.001
Respiratory rate rest, T0 (breaths/min)	15.01 ± 2.24	15.34 ± 2.70	
Respiratory rate rest, T1 (breaths/min)	13.06 ± 2.66	15.26 ± 2.63	
Respiratory rate rest, ΔT1–T0 (breaths/min)	−1.95 (−2.33 to −1.58)	−0.08 (−0.33 to 0.17)	−1.87 (−2.31 to −1.43); P < 0.001
ERQ reappraisal (1–7), T0	4.11 ± 0.87	3.57 ± 0.81	
ERQ reappraisal (1–7), T1	4.85 ± 0.93	3.84 ± 0.93	
ERQ reappraisal, ΔT1–T0	+0.74 (0.58 to 0.91)	+0.27 (0.14 to 0.39)	+0.48 (0.27 to 0.68); *P* < 0.001
SCS total (1–5), T0	3.02 ± 0.62	3.06 ± 0.56	
SCS total (1–5), T1	3.44 ± 0.62	3.16 ± 0.58	
SCS total, ΔT1–T0	+0.42 (0.33 to 0.51)	+0.10 (0.03 to 0.16)	+0.32 (0.21 to 0.43); *P* < 0.001
MAIA‑2 total (0–5), T0	2.69 ± 0.62	2.72 ± 0.63	
MAIA‑2 total (0–5), T1	3.02 ± 0.72	2.75 ± 0.61	
MAIA‑2 total, ΔT1–T0	+0.33 (0.23 to 0.43)	+0.04 (−0.04 to 0.11)	+0.29 (0.17 to 0.42); *P* < 0.001

Values/statistics as in Table 2. Abbreviations: RMSSD, root mean square of successive differences; HFnu, high‑frequency power in normalized units; ERQ, Emotion Regulation Questionnaire; SCS, Self‑Compassion Scale; MAIA‑2, Multidimensional Assessment of Interoceptive Awareness (version 2).

### Stress reactivity substudy

**Fig 2:**
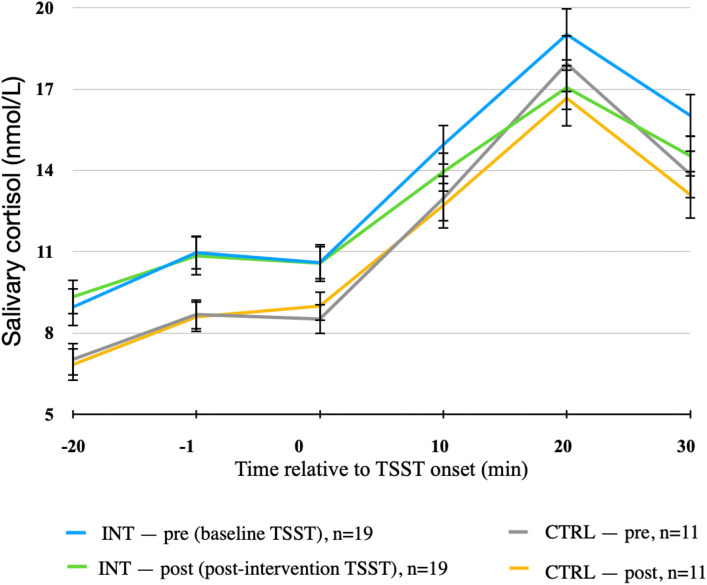
TSST cortisol trajectories before and after the intervention. The figure shows mean (±SEM) salivary cortisol concentrations at t = −20, −1, 0, + 10, + 20, and +30 min relative to TSST onset. TSST onset was defined as the start of the speech task; the preparation period occurred from t = −5 to 0, the speech task from t = 0 to 5, the arithmetic task from t = 5 to 10, and the recovery period after t = 10. Trajectories are shown for the intervention group (INT; n = 19) and active control group (CTRL; n = 11) at the baseline and post-intervention TSST sessions. Error bars indicate SEM across participants. The sampling window captures the expected post-onset rise and peak latency of salivary cortisol following acute psychosocial stress, and the final time point reflects early recovery rather than late recovery. Because no + 60 or +90 min samples were collected, the figure does not characterize late recovery or HPA-axis shut-off dynamics.

Participants in the TSST subsample completed serial saliva sampling at t = −20, −1, 0, + 10, + 20, and +30 min relative to TSST onset, both pre- and post-intervention. The cortisol trajectory showed the expected post-onset rise with a peak at approximately +20 min in nearly all sessions (Fig 2), indicating successful stress induction and capture of peak-latency dynamics within the collected window. Because sampling ended at +30 min relative to TSST onset, late recovery was not assessed. Summary indices therefore focused on peak cortisol and baseline-corrected AUCi within the collected −1 to +30 min window (Table 5).

**Table 5:** Trier Social Stress Test (TSST) Peak and AUCi cortisol reactivity within the collected sampling window.

Outcome	Intervention (n = 19)	Control (n = 11)	Between‑group difference in change (INT–CTRL)
Peak salivary cortisol during TSST, pre (nmol/L)	19.02 ± 4.22	17.94 ± 3.87	
Peak salivary cortisol during TSST, post (nmol/L)	17.06 ± 3.67	16.67 ± 3.53	
Δpost–pre (nmol/L)	−1.96 (−2.58 to −1.34)	−1.27 (−2.20 to −0.34)	−0.69 (−1.71 to 0.33); P = 0.178
TSST cortisol AUCi (−1 to +30 min), pre (nmol/L·h)	2.39 ± 0.91	2.67 ± 0.90	
TSST cortisol AUCi (−1 to +30 min), post (nmol/L·h)	1.84 ± 0.79	2.45 ± 0.84	
Δpost–pre (nmol/L·h)	−0.55 (−0.97 to −0.14)	−0.23 (−0.61 to 0.16)	−0.33 (−0.93 to 0.27); P = 0.271

Serial TSST saliva sampling was performed at t = −20, −1, 0, + 10, + 20, and +30 min relative to TSST onset (t = 0, defined as the start of the speech task). The active stressor ended at approximately t = +10 min after the speech and mental-arithmetic tasks; therefore, the + 20 and +30 min samples correspond to approximately 10 and 20 min after task cessation, respectively. Descriptive values at each session are untransformed raw-scale mean ± SD; within-group changes and between-group differences are untransformed raw-scale mean changes with 95% CI. The rightmost column reports the between-group difference in mean change (INT–CTRL) with 95% CI and two-sided P values from ordinary least squares on individual Δ(post–pre). Peak cortisol was defined as the maximum across post-onset samples (t = 0 to +30 min). AUCi was computed by trapezoidal integration over t = −1 to +30 min and baseline-corrected to the t = −1 min sample (Pruessner AUCi approach). These indices quantify peak-magnitude and baseline-corrected reactivity within the collected sampling window. No samples were collected at +60 or +90 min, so late recovery and HPA-axis shut-off dynamics were not characterized. TSST peak cortisol was analyzed on the natural-log scale in the inferential mixed-effects model (S2 Table). Baseline-corrected TSST cortisol AUCi was evaluated using the untransformed raw-scale OLS change-score comparison shown in this table. The descriptive peak and AUCi values are reported on the original units (nmol/L and nmol/L·h).

Baseline-corrected TSST cortisol AUCi over t = −1 to +30 min decreased from pre to post in both groups; however, the between-group difference in change was not statistically significant (ΔINT–CTRL = −0.33 nmol/L·h, 95% CI, −0.93 to 0.27; P = 0.271). Peak cortisol within t = 0 to +30 min also decreased from pre to post in both groups, with no statistically significant between-group difference in change (ΔINT–CTRL = −0.69 nmol/L, 95% CI, −1.71 to 0.33; P = 0.178). Thus, the substudy provides little evidence that INT altered peak-magnitude or baseline-corrected AUCi cortisol reactivity within the collected sampling window.

### Exposure, fidelity, and adherence

Attendance was high and comparable (INT 7.43 ± 0.73 vs CTRL 7.63 ± 0.61 of 8 sessions; S3 Table). Treatment differentiation was robust: coder‑rated minutes per session indicated that INT allocated substantially more time to affective‑social practice (34.6 vs 5.5 min), breath/HRV training (32.8 vs 4.4 min), reappraisal (23.0 vs 12.1 min), and mindfulness (24.8 vs 13.9 min), whereas CTRL emphasized psychoeducation (101.0 vs 19.8 min). Home‑practice minutes/week mirrored this pattern (e.g., breath/HRV 66.5 vs 1.2 min).

### Safety, blinding, and assay quality control

Adverse events were infrequent and similar between groups (3/30 [10%] each; S4 Table). Participant guessing of assignment at T1 approximated chance (INT 50%, CTRL 47%). Laboratory performance was stable and analyte‑specific: mean (range) intra‑/inter‑assay CVs were 4.8% (3.9–5.9)/ 8.1% (6.7–9.4) for cortisol, 5.6% (4.4–6.9)/ 9.3% (7.9–10.8) for cortisone, and 3.7% (2.9–4.8)/ 7.1% (6.1–8.6) for α‑amylase. Procedures followed the prespecified quality controls (timing windows, multi‑day sampling, and batch/plate checks).

## Discussion

### Principal findings

In this randomized, active‑controlled trial, an integrative group intervention designed to target both autonomic regulation (slow breathing/HRV‑biofeedback) and cognitive–socio‑affective skills yielded improvements in multi-day diurnal HPA-axis output and sympathetic-linked indices in daily life. Across three sampling days per wave, cortisol AUCg decreased relative to the dose-matched control, with robust reductions in daily cortisol output (AUCg) and hair cortisol, while the wake-to-bed diurnal slope became less negative. We decomposed this finding by inspecting the absolute awakening and bedtime concentrations. The less-negative slope was not caused by an elevation in bedtime cortisol; bedtime values decreased in INT. Rather, awakening and early-morning cortisol values decreased more strongly than bedtime values, reducing the morning-to-evening amplitude from which the wake-to-bed slope is calculated. Therefore, the slope result is best interpreted as a change in profile geometry within a lower overall daily cortisol profile, not as favorable slope normalization. Because flatter slopes have been associated with adverse outcomes in prior meta-analyses, slope findings should be interpreted cautiously and should not be used as evidence of improvement [1]. Salivary cortisone—assayed alongside cortisol—showed parallel patterns, and salivary α‑amylase declined, aligning with the intervention’s autonomic focus. Hair cortisol concentration was lower in the proximal 1-cm segment collected at extended follow-up, indicating lower tissue glucocorticoid levels during the month immediately preceding the T3 visit. This finding should not be interpreted as a continuous or cumulative measure of glucocorticoid exposure across the entire T1–T3 interval, because the interval between the T1 and T3 proximal segments was not assayed. Thus, the 6-month follow-up denotes the timing of the T3 collection visit rather than an integrated 6-month retrospective window. Taken together, the data indicate axis‑concordant physiological change in daily life that is mechanism‑consistent with the skills trained. As per the preregistered plan, we optimized measurement fidelity (multi‑day sampling; time‑stamped CAR windows; actigraphy; inclusion of cortisone and sAA) and analyzed outcomes with mixed‑effects models, limiting analytic flexibility.

### Interpretation in the context of prior literature

Behavioral stress‑reduction trials have historically reported heterogeneous effects on salivary cortisol, partly due to measurement variability (sampling windows, number of days, adherence checks). Our findings are directionally consistent with the most rigorous segments of that literature: a systematic review of RCTs emphasized inconsistent cortisol effects in earlier trials and the need for standardized, multi‑day protocols and prespecified metrics, which the present study followed [14]. Moreover, reliability work demonstrates that diurnal features differ markedly in day‑to‑day stability (ICC range ~0.00–0.75), with total output (AUCg) generally more stable than the awakening rise; this underscores why AUCg often yields more stable estimates than CAR across days [15]. Notably, despite the higher day-to-day variability typically observed for CAR, we found a significant between-group reduction in CAR (AUCi) in the intervention arm, alongside reductions in daily cortisol output (AUCg).

Adding cortisone likely improved endocrine signal‑to‑noise: cortisone tracks serum free cortisol more closely than salivary cortisol, particularly at low concentrations or when CBG shifts are present. The parallel cortisone changes observed here therefore strengthen inference that the intervention altered systemic HPA exposure rather than assay artifacts [4,19,20]. The HCC reduction at follow-up mirrors large-scale evidence that contemplative mental training over months can reduce hair glucocorticoids [7]. In the present trial, however, the proximal 1-cm segment indexes the month before each collection point. Therefore, the T3 HCC result should be interpreted as lower proximal-segment hair cortisol at the 6-month follow-up collection, not as a continuous measure of the entire follow-up interval.

On sympathetic outputs, the sAA decrease aligns with meta‑analytic and mechanistic evidence for HRV‑biofeedback and resonance‑frequency breathing increasing vagal tone and reducing stress symptoms. While HRVB’s downstream influence on cortisol is inconsistent, autonomic benefits are robust— consistent with our two-axis pattern of concurrent reductions in sAA and glucocorticoid indices [10,21,22].

Finally, the TSST substudy showed trends but no clear between-group difference in peak/AUCi cortisol reactivity within the collected sampling window. Because sampling extended only to +30 min relative to TSST onset, the substudy captured the expected post-onset rise and peak-latency/early post-task phase but did not characterize late recovery. Thus, the substudy does not show evidence that INT altered peak-magnitude cortisol reactivity or baseline-corrected AUCi within the collected window, and HPA-axis recovery or shut-off dynamics remain uncharacterized because sampling did not extend to +60 or +90 min. In the ReSource Project, social-affective/socio-cognitive training modules attenuated TSST cortisol by up to ~50%, whereas attention-focused practice did not. Null or attenuated effects here may reflect (i) smaller sample/power; (ii) procedural or timing moderators; (iii) sex-hormone composition, such as oral contraceptive use; and (iv) potential habituation to repeated psychosocial stress exposure, which can attenuate HPA-axis reactivity on re-administration and reduce sensitivity to detect between-arm effects [9,19].

### Mechanistic considerations

The intervention was designed to engage complementary levers of stress regulation. First, slow diaphragmatic breathing near ~0.1 Hz with HRV‑biofeedback recruits baroreflex–vagal mechanisms, increasing HRV and reducing sympathetic outflow—consistent with observed sAA decreases and HRV improvements as proximal targets [10,21,22]. Second, socio-affective training may buffer social-evaluative threat, a primary driver of HPA activation in the TSST, and therefore could contribute to diurnal load reduction even if between-group shifts in TSST peak/AUCi cortisol within the collected window were not statistically significant [9]. Third, cognitive reappraisal/stress-mindset may enhance appraisal efficiency and emotion-regulation target engagement; endocrine effects are typically context-dependent, which may help explain the dissociation between reduced daily-life glucocorticoid output/proximal-segment hair cortisol and the absence of a clear between-group effect on TSST peak/AUCi cortisol reactivity within the collected sampling window [23,24].

The coherent changes observed across multiple glucocorticoid indices (AUCg, CAR, and HCC) are mechanistically plausible. CAR and diurnal output reflect partially distinct generators; a recent meta‑analysis found CAR unrelated to acute stress reactivity, underscoring that interventions lowering daily load need not alter the awakening surge [24].

### Measurement choices and their implications

Three features of the design likely amplified assay sensitivity. (i) Three-day diurnal sampling per wave improves reliability relative to single‑day protocols [15]. (ii) Objective adherence (actigraphy/time‑stamps) reduces CAR misclassification, a major bias when awakening samples are delayed [2]. (iii) Incorporating cortisone and sAA provided two‑axis coverage, important because sympathetic indices often show faster, larger changes to training than cortisol, and because cortisone improves inference about systemic HPA activity [4,11,20]. The TSST protocol followed established practice for eliciting robust HPA responses and captured the expected peak-latency window, yet individual-difference moderators (sex, oral-contraceptive status, and time of day) can attenuate effects. Because sampling ended at +30 min, our mixed TSST results should be interpreted as peak/AUCi reactivity findings rather than as evidence about late recovery dynamics [18,24,25].

The decomposition of the slope endpoint also illustrates why wake-to-bed slopes should be interpreted alongside their component concentrations: a slope can become less negative when morning cortisol decreases substantially, even if evening cortisol does not increase.

### Clinical and translational relevance

Participants entered the trial with subclinical–moderate anxiety/depression symptom burden (Table 1). The present report focuses on physiological endpoints; therefore, we do not make claims about longitudinal changes in depressive/anxiety symptoms or perceived stress based on the current Results section. Future work should evaluate whether the observed endocrine and autonomic shifts translate into clinically meaningful symptom trajectories. Importantly, the active control strengthens causal attribution by mitigating expectancy and contact-time confounds; reliance on waitlist controls is known to inflate psychotherapy effect sizes [26].

### Strengths and limitations

This study has several strengths. First, the trial followed a prespecified statistical analysis plan and used a parallel-group design with a dose-matched active comparator. Second, the study used multi-day diurnal saliva sampling with objective adherence checks, which reduces timing-related bias in ambulatory cortisol assessment. Third, the endocrine panel combined cortisol, cortisone, salivary α-amylase, and hair cortisol concentration, allowing complementary assessment of HPA-axis and sympathetic-linked indices. Fourth, proximal target-engagement measures, including HRV, respiratory rate, emotion regulation, self-compassion, and interoceptive awareness, were assessed to support future mediation analyses. Fifth, treatment fidelity and differentiation procedures documented whether the intended intervention ingredients were delivered.

Several limitations should also be considered. The sample size limited power for some secondary analyses, particularly the Trier Social Stress Test substudy and potential moderator or mediator tests. In addition, the TSST sampling schedule ended at +30 min relative to task onset and did not include +60 or +90 min samples, so late HPA-axis recovery and shut-off dynamics could not be evaluated. Potential cluster effects related to therapy group or therapist were modeled, but the number of clusters was small. Generalizability is limited to adult volunteers with subclinical to moderate anxiety/depression symptoms. At-home saliva collection may retain residual preanalytic variability despite objective timing controls. The study did not include immuno-inflammatory endpoints, such as salivary IL-1β, IL-6, TNF-α, or systemic markers such as CRP, which could have broadened mechanistic inference. Finally, because no dedicated coping-style or coping-strategy inventory was administered, the present data support changes in emotion-regulation, self-compassion, interoceptive awareness, and socio-affective target engagement, but they do not permit direct inference about coping styles or coping strategies.

### Future directions

Replication with multi‑site samples and a priori powered moderator/mediator tests is warranted. Mechanistically, integrating immuno‑inflammatory markers (e.g., IL‑6, CRP), wearable physiology, and EMA‑captured skill use on sampling days should clarify dose–response and within‑day coupling, given emerging evidence that momentary stress/affect covaries with near‑time cortisol when protocols are rigorous [27]. Future HCC studies should add later hair collection points and, where feasible, predefined segmental hair analyses to test whether lower proximal-segment hair cortisol persists across follow-up intervals. Such designs would better distinguish repeated evidence of lower tissue glucocorticoid levels near each collection from continuous cumulative exposure across the full follow-up period. Finally, stratified analyses by sex/OCs and time‑of‑day could optimize TSST sensitivity and adjudicate for whom social‑affective components most strongly modulate reactivity.

## Conclusions

In this randomized, assessor- and laboratory-blinded trial with a dose-matched active control, an 8-week integrative group intervention combining social-affective skills training, slow-breathing/HRV-biofeedback, and cognitive reappraisal reduced the prespecified primary endpoint of three-day averaged daily salivary cortisol output. This reduction was greater in the intervention group than in the control group and was maintained at short follow-up. Salivary cortisone and salivary α-amylase AUCg also decreased more in the intervention group, indicating lower daily-life endocrine and sympathetic-linked output under active-control conditions. Hair cortisol concentration was lower in the proximal 1-cm segments collected at the end of treatment and at the 6-month follow-up visit, with each segment indexing approximately the month before collection rather than the entire follow-up interval. The wake-to-bed diurnal cortisol slope became less negative and should not be interpreted as independent evidence of physiological improvement; profile decomposition showed that this change reflected a larger reduction in awakening and early-morning cortisol rather than an increase in bedtime cortisol. Concurrent improvements in HRV, resting respiratory rate, reappraisal, self-compassion, and interoceptive awareness support the intervention’s target-engagement rationale.

The findings indicate that a mechanistically specified, multicomponent behavioral program can reduce selected daily-life and tissue-level stress-biomarker indices when tested against an active comparator under high-fidelity endocrine measurement. The pattern of findings—reduced daily cortisol output, lower proximal-segment hair cortisol in the month preceding follow-up collection points, reduced CAR AUCi, and little evidence of altered TSST peak/AUCi reactivity within the collected −1 to +30 min sampling window—is consistent with the view that different HPA/SNS indices capture partly distinct regulatory processes. Because the TSST protocol did not include +60 or +90 min samples, late recovery and HPA-axis shut-off dynamics remain uncharacterized. Methodologically, the study supports the value of multi-day sampling, objective timing, cortisone co-assay, and two-axis HPA/SNS panels when endocrine outcomes are primary.

Larger multi-site trials with longer follow-up, cardiometabolic and immuno-inflammatory endpoints, and adequately powered mediator and moderator analyses are needed to test durability, clinical translation, and mechanisms. Future studies should determine whether changes in autonomic and cognitive-affective target engagement mediate reductions in daily cortisol output, salivary α-amylase, and proximal-segment hair cortisol.

## Supporting information

S1 Dataset De-identified individual participant-level dataset (English) with accompanying codebook and endpoint derivation notes. (XLSX)

S2 Dataset Dataset deidentified English v5 metadata clean. (XLSX)

S1 Fig Study timeline and assessment schedule across T0, T1, T2, and T3, including diurnal saliva sampling days, TSST substudy schedule, and hair collection time points. (PDF)

S1 Table Prespecified mixed-effects ITT models for primary diurnal endocrine endpoints (cortisol AUCg, CAR AUCi, diurnal slope). (DOCX)

S2 Table Prespecified mixed-effects ITT models for additional endocrine endpoints and supporting analyses. (DOCX)

S3 Table Process measures, fidelity/differentiation coding, attendance, and home-practice dose. (DOCX)

S4 Table Safety, expectancy/blinding checks, and assay QC metrics. (DOCX)

S5 Table Sensitivity analysis using conventional mixed-design repeated-measures ANOVA (Time×Group interaction). (DOCX)

S6 Table Absolute salivary cortisol concentrations across the diurnal sampling profile underlying the diurnal slope finding. (DOCX)

S1 File CONSORT Checklist. (DOCX)

S2 File Trial protocol and prespecified statistical analysis plan (SAP), original Russian version. (PDF)

S3 File Trial protocol and prespecified statistical analysis plan (SAP), English translation. (PDF)
