# Supplementary material for: Integrative group psychotherapy reduces daily cortisol output and hair cortisol: A randomized active‑controlled trial with multi‑day profiling
Source: PLoS One. 2026 Jul 23;21(7):e0352095. doi: 10.1371/journal.pone.0352095 (PMC13395371; doi:10.1371/journal.pone.0352095)
Supplement: S1 Fig — (PDF) [file pone.0352095.s003.pdf]

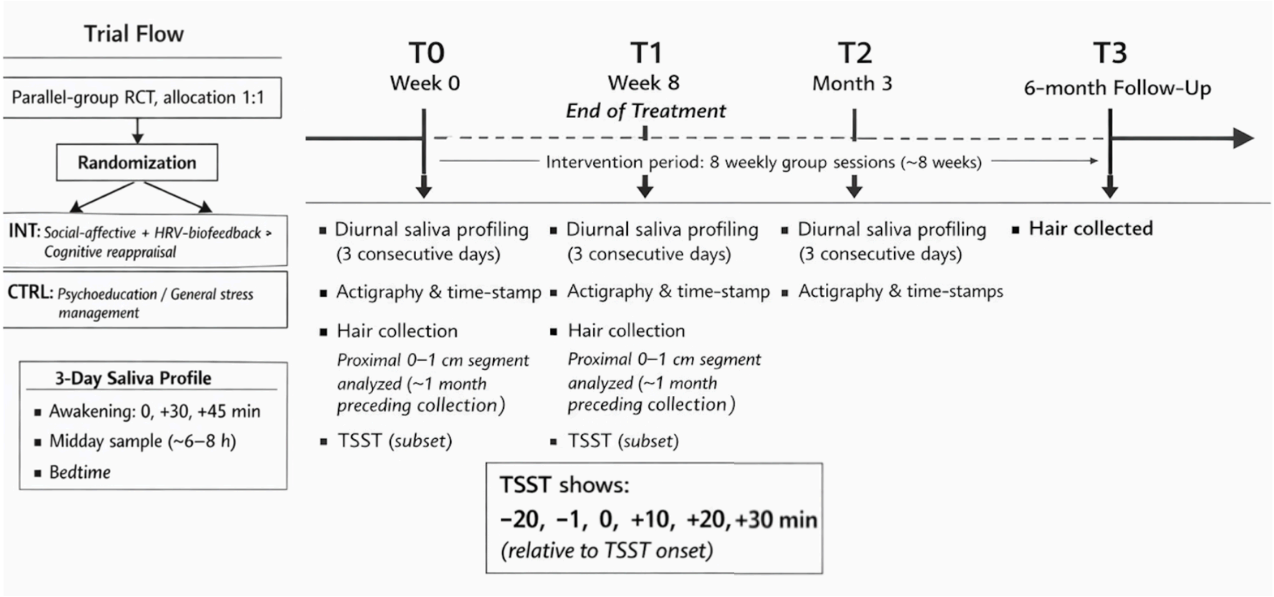

S1 Fig. Study design and assessment timeline. Parallel-group randomized, active-controlled trial (1:1) with eight weekly group sessions (INT vs CTRL). Diurnal saliva profiling (three consecutive sampling days per wave) was performed at T0, T1, and T2; hair was collected at T0, T1, and T3 (proximal 1-cm segment; ≈1-month exposure immediately preceding each collection). The TSST substudy was conducted pre- and post-intervention.
