# Supplementary material for: Integrative group psychotherapy reduces daily cortisol output and hair cortisol: A randomized active‑controlled trial with multi‑day profiling
Source: PLoS One. 2026 Jul 23;21(7):e0352095. doi: 10.1371/journal.pone.0352095 (PMC13395371; doi:10.1371/journal.pone.0352095)
Supplement: S1 Table — (DOCX) [file pone.0352095.s004.docx]

**Appendix**

**Table S1.** ITT mixed‑effects (log scale unless noted): prespecified primary and salivary secondary outcomes

| Outcome (model) | Contrast | β (SE) | 95% CI | P value | INT vs CTRL ratio‑of‑change |
| --- | --- | --- | --- | --- | --- |
| log(AUCg cortisol) | T1×INT | −0.223 (0.045) | −0.311, −0.136 | <0.001 | 0.80 (−20.0%) |
|  | T2×INT | −0.249 (0.049) | −0.346, −0.152 | <0.001 | 0.78 (−22.1%) |
| log(AUCg cortisone) | T1×INT | −0.232 (0.068) | −0.367, −0.097 | <0.001 | 0.79 (−20.7%) |
|  | T2×INT | −0.269 (0.073) | −0.412, −0.126 | <0.001 | 0.76 (−23.6%) |
| log(CAR AUCi, cortisol) | T1×INT | −0.466 (0.118) | −0.698, −0.234 | <0.001 | 0.63 (−37.2%) |
|  | T2×INT | −0.511 (0.131) | −0.770, −0.252 | <0.001 | 0.60 (−40.0%) |
| log(AUCg sAA) | T1×INT | −0.130 (0.045) | −0.218, −0.042 | 0.004 | 0.88 (−12.2%) |
|  | T2×INT | −0.148 (0.049) | −0.244, −0.052 | 0.002 | 0.86 (−13.8%) |
| Diurnal slope (nmol/L per h, raw; wake-to-bed endpoint) | T1×INT | +0.067 (0.022) | +0.024, +0.110 | 0.002 | — |
|  | T2×INT | +0.062 (0.023) | +0.017, +0.107 | 0.006 | — |

*Model footnote:* Fixed: time (T0, T1, T2), group, time×group; Random: intercepts for participant and for therapist nested in therapy group; Covariates: awakening time, sampling‑window adherence, caffeine/smoking flags, plate/batch, freeze–thaw; log‑transform for hormones; Satterthwaite df; two‑sided tests; FDR at q=0.05 for secondary families. For diurnal cortisol slope, the endpoint was calculated uniformly as the wake-to-bed slope for each sampling day: (bedtime cortisol − awakening cortisol) divided by the elapsed hours between the actual WAK0 and EVE sample timestamps. The three day-level slopes were averaged to obtain the participant-wave endpoint. WAK30 and WAK45 samples were not used in the slope calculation to avoid conflation with the cortisol awakening response. Model coefficients are reported on the model scale shown in the Outcome column. For log-transformed outcomes, the ratio-of-change column provides back-transformed effects; raw-scale descriptive summaries are reported in the main manuscript tables. Diurnal slope was modeled on the raw scale, so no back-transformed ratio-of-change is applicable.
