# Supplementary material for: Integrative group psychotherapy reduces daily cortisol output and hair cortisol: A randomized active‑controlled trial with multi‑day profiling
Source: PLoS One. 2026 Jul 23;21(7):e0352095. doi: 10.1371/journal.pone.0352095 (PMC13395371; doi:10.1371/journal.pone.0352095)
Supplement: S2 Table — (DOCX) [file pone.0352095.s005.docx]

**Table S2.** ITT mixed‑effects: hair cortisol concentration and TSST substudy

| Outcome (model) | Contrast | β (SE) | 95% CI | P value | INT vs CTRL ratio‑of‑change |
| --- | --- | --- | --- | --- | --- |
| log(HCC, pg/mg) | T1×INT | −0.143 (0.041) | −0.223, −0.064 | <0.001 | 0.87 (−13.3%) |
|  | T3×INT | −0.201 (0.052) | −0.303, −0.099 | <0.001 | 0.82 (−18.2%) |
| log(TSST peak cortisol) | post×INT | −0.041 (0.030) | −0.100, 0.018 | 0.17 | 0.96 (−4.0%) |

*Footnotes:* HCC and TSST peak cortisol were modeled on the natural-log scale; ratio-of-change values are back-transformed from the model coefficients. Descriptive HCC values in Table 3 and descriptive TSST peak/AUCi values in Table 5 are untransformed raw-scale values. HCC rows refer to hair cortisol concentration measured from the proximal 0–1 cm segment at each collection. T3 denotes the 6-month follow-up hair collection visit; the corresponding proximal segment indexes approximately the month immediately preceding that visit and should not be interpreted as a 6-month retrospective segment or as a continuous measure of the full T1–T3 interval. TSST peak cortisol was defined within the collected post-onset window as the maximum value across t = 0 to +30 min; no TSST samples were collected at +60 or +90 min, so late recovery and HPA-axis shut-off dynamics were not characterized.
