# Supplementary material for: Integrative group psychotherapy reduces daily cortisol output and hair cortisol: A randomized active‑controlled trial with multi‑day profiling
Source: PLoS One. 2026 Jul 23;21(7):e0352095. doi: 10.1371/journal.pone.0352095 (PMC13395371; doi:10.1371/journal.pone.0352095)
Supplement: S3 Table — (DOCX) [file pone.0352095.s006.docx]

**Table S3.** Treatment Exposure, Fidelity, and Adherence

| Process metric | Intervention | Control |
| --- | --- | --- |
| Sessions attended (of 8), mean ± SD | 7.43 ± 0.73 | 7.63 ± 0.61 |
| Session content minutes per session: Affective social | 34.6 | 5.5 |
| Session content minutes per session: Reappraisal | 23.0 | 12.1 |
| Session content minutes per session: Breath HRV | 32.8 | 4.4 |
| Session content minutes per session: Mindfulness | 24.8 | 13.9 |
| Session content minutes per session: Psychoeducation | 19.8 | 101.0 |
| Home practice minutes/week: Breath HRV | 66.5 | 1.2 |
| Home practice minutes/week: Affective social | 57.5 | 0.3 |
| Home practice minutes/week: Reappraisal | 51.4 | 1.3 |
| Home practice minutes/week: Mindfulness | 61.3 | 24.1 |
| Home practice minutes/week: Psychoeducation | 9.9 | 57.6 |

*Footnotes:* Session content minutes are coder‑rated (treatment differentiation); home practice minutes/week are self‑reported averages across the 8‑week course. Abbreviation: HRV, heart rate variability.
