# Supplementary material for: Integrative group psychotherapy reduces daily cortisol output and hair cortisol: A randomized active‑controlled trial with multi‑day profiling
Source: PLoS One. 2026 Jul 23;21(7):e0352095. doi: 10.1371/journal.pone.0352095 (PMC13395371; doi:10.1371/journal.pone.0352095)
Supplement: S4 Table — (DOCX) [file pone.0352095.s007.docx]

**Table S4.** Safety, Blinding, and Assay Quality Control

| Safety and assay QC | Intervention | Control |
| --- | --- | --- |
| Participants with ≥1 adverse event, n (%) | 3/30 (10) | 3/30 (10) |
| NEQ total score (mean) | 3.4 | 4.0 |
| Correct guess of assignment at T1, n (%) | 15/30 (50) | 14/30 (47) |
| Assay CV% (intra / inter), CORT | 4.8 / 8.1 |  |
| Assay CV% (intra / inter), CORTISONE | 5.6 / 9.3 |  |
| Assay CV% (intra / inter), SAA | 3.7 / 7.1 |  |

*Footnotes:* Values are means across plates and QC levels; ranges were cortisol 3.9–5.9 / 6.7–9.4, cortisone 4.4–6.9 / 7.9–10.8, and sAA 2.9–4.8 / 6.1–8.6 (intra‑/inter‑assay CV%). Abbreviations: NEQ, Negative Effects Questionnaire; CV, coefficient of variation; CORT, salivary cortisol; SAA, salivary α‑amylase.
