# Supplementary material for: Integrative group psychotherapy reduces daily cortisol output and hair cortisol: A randomized active‑controlled trial with multi‑day profiling
Source: PLoS One. 2026 Jul 23;21(7):e0352095. doi: 10.1371/journal.pone.0352095 (PMC13395371; doi:10.1371/journal.pone.0352095)
Supplement: S5 Table — (DOCX) [file pone.0352095.s008.docx]

**Table S5.** Sensitivity analysis using conventional mixed‑design repeated‑measures ANOVA (Time×Group interaction).

| Outcome | Time levels | N | F (df1, df2) | P value | ηp² |
| --- | --- | --- | --- | --- | --- |
| Daily cortisol AUCg (nmol/L·h) | T0, T1, T2 | 60 | 53.36 (2, 116) | 3.70e‑17 | 0.479 |
| Daily cortisone AUCg (nmol/L·h) | T0, T1, T2 | 60 | 49.96 (2, 116) | 2.24e‑16 | 0.463 |
| CAR AUCi (cortisol; nmol/L·h) | T0, T1, T2 | 60 | 10.71 (2, 116) | 5.40e‑05 | 0.156 |
| Daily sAA AUCg (U/mL·h) | T0, T1, T2 | 60 | 21.50 (2, 116) | 1.14e‑08 | 0.270 |
| Diurnal cortisol slope (nmol/L per h) | T0, T1, T2 | 60 | 4.96 (2, 116) | 8.60e−03 | 0.079 |
| Hair cortisol concentration (HCC; pg/mg) | T0, T1, T3 | 60 | 68.11 (2, 116) | 2.73e‑20 | 0.540 |
| RMSSD rest (ms) | T0, T1 | 60 | 41.62 (1, 58) | 2.43e08 | 0.418 |
| HFnu rest | T0, T1 | 60 | 60.19 (1, 58) | 1.56e10 | 0.509 |
| Respiratory rate rest (breaths/min) | T0, T1 | 60 | 72.43 (1, 58) | 8.59e12 | 0.555 |

*Footnotes:* Conventional mixed‑design repeated‑measures ANOVA with Time as the within‑subject factor (T0 baseline; T1 end of treatment; T2 ~3 months follow‑up; for hair cortisol: T3 ~6 months follow‑up) and Group as the between‑subject factor (INT vs CTRL). Reported values are Time×Group interaction F tests. N indicates the number of participants with complete outcome values across the listed time levels for that endpoint. ηp² denotes partial eta‑squared effect size. Two‑sided P values. The diurnal cortisol slope row uses the wake-to-bed endpoint calculated for each sampling day as (bedtime cortisol − awakening cortisol) divided by elapsed hours between the actual WAK0 and EVE sample timestamps, then averaged across the three sampling days. Because a less-negative wake-to-bed slope can result from reduced awakening cortisol rather than elevated bedtime cortisol, this Time×Group effect is reported descriptively and is not interpreted as favorable slope normalization. The RM-ANOVA sensitivity analysis used the same transformation rules as the primary LMMs: cortisol AUCg, cortisone AUCg, sAA AUCg, CAR AUCi, and HCC were analyzed on the natural-log scale, whereas diurnal cortisol slope, RMSSD, HFnu, and respiratory rate were analyzed on the raw scale. Units in the Outcome column identify the original descriptive metric reported in the manuscript, not necessarily the model scale used for the F test.
