# Supplementary material for: Integrative group psychotherapy reduces daily cortisol output and hair cortisol: A randomized active‑controlled trial with multi‑day profiling
Source: PLoS One. 2026 Jul 23;21(7):e0352095. doi: 10.1371/journal.pone.0352095 (PMC13395371; doi:10.1371/journal.pone.0352095)
Supplement: S6 Table — (DOCX) [file pone.0352095.s009.docx]

**S6 Table.** Absolute awakening and bedtime cortisol concentrations underlying the diurnal slope finding

| **Sample** | **Group** | **T0** | **T1** | **T2** | **ΔT1–T0** | **ΔT2–T0** |
| --- | --- | --- | --- | --- | --- | --- |
| Awakening WAK0 | INT | 12.00 ± 3.17 | 9.42 ± 2.38 | 9.03 ± 2.34 | −2.57 (−3.08 to −2.07) | −2.97 (−3.52 to −2.42) |
| Awakening WAK0 | CTRL | 13.24 ± 2.62 | 11.97 ± 2.26 | 11.68 ± 2.32 | −1.27 (−1.64 to −0.89) | −1.56 (−1.98 to −1.14) |
| Bedtime/EVE | INT | 2.49 ± 0.94 | 1.91 ± 0.76 | 1.87 ± 0.59 | −0.58 (−0.80 to −0.36) | −0.61 (−0.82 to −0.40) |
| Bedtime/EVE | CTRL | 2.21 ± 0.82 | 2.10 ± 0.90 | 2.19 ± 0.92 | −0.11 (−0.32 to 0.09) | −0.02 (−0.26 to 0.22) |

*Footnotes:* Values are untransformed raw salivary cortisol concentrations in nmol/L, reported as mean ± SD across participant-level wave means. WAK0 and EVE values were averaged across the three sampling days within each participant and wave before group-level summaries were computed. Changes are mean change with 95% CI. The between-group difference in ΔT1–T0 was −1.31 nmol/L for WAK0 (95% CI, −1.92 to −0.69; P<0.001) and −0.47 nmol/L for EVE (95% CI, −0.77 to −0.17; P=0.003). Thus, the less-negative wake-to-bed slope was not driven by elevated bedtime cortisol.

| **Sample** | **Group** | **T0** | **T1** | **T2** |
| --- | --- | --- | --- | --- |
| WAK0 | INT | 12.00 ± 3.17 | 9.42 ± 2.38 | 9.03 ± 2.34 |
| WAK0 | CTRL | 13.24 ± 2.62 | 11.97 ± 2.26 | 11.68 ± 2.32 |
| WAK30 | INT | 15.99 ± 3.09 | 11.91 ± 2.46 | 11.29 ± 2.36 |
| WAK30 | CTRL | 16.70 ± 3.01 | 15.31 ± 2.70 | 14.95 ± 2.34 |
| WAK45 | INT | 18.32 ± 3.17 | 11.82 ± 2.02 | 11.12 ± 2.29 |
| WAK45 | CTRL | 18.90 ± 3.09 | 16.24 ± 2.85 | 15.70 ± 2.72 |
| MID | INT | 5.22 ± 2.32 | 4.38 ± 1.91 | 4.18 ± 1.79 |
| MID | CTRL | 6.30 ± 2.16 | 5.85 ± 2.05 | 5.55 ± 2.04 |
| EVE | INT | 2.49 ± 0.94 | 1.91 ± 0.76 | 1.87 ± 0.59 |
| EVE | CTRL | 2.21 ± 0.82 | 2.10 ± 0.90 | 2.19 ± 0.92 |
