# Supplementary material for: Integrative group psychotherapy reduces daily cortisol output and hair cortisol: A randomized active‑controlled trial with multi‑day profiling
Source: PLoS One. 2026 Jul 23;21(7):e0352095. doi: 10.1371/journal.pone.0352095 (PMC13395371; doi:10.1371/journal.pone.0352095)
Supplement: S1 File — (DOCX) [file pone.0352095.s010.docx]

|  | Section/topic | No | CONSORT 2025 checklist item description | Reported on page no. |
| --- | --- | --- | --- | --- |
|  | **Title and abstract** | | |  |
|  | Title and structured abstract | 1a | Identification as a randomised trial | p.1 – Title; design line “Parallel‑group, superiority, randomized, active‑controlled trial (allocation ratio 1:1)” |
|  |  | 1b | Structured summary of the trial design, methods, results, and conclusions | p.2 – Abstract (structured: Background, Methods, Results, Conclusions) |
|  | **Open science** | | |  |
|  | Trial registration | 2 | Name of trial registry, identifying number (with URL) and date of registration | pp.1–2, 4–5 – Title page (“Trial registration: ClinicalTrials.gov NCT06863948; date of registration: 07 Mar 2025; first participant enrolled: 9 Mar 2025”); Abstract; Methods – Study Design and Oversight |
|  | Protocol and statistical analysis plan | 3 | Where the trial protocol and statistical analysis plan can be accessed | pp.1, 4–5 – Title page (“Protocol & SAP access: Full protocol and prespecified SAP available”); Methods – Study Design and Oversight; Supporting Information – S2/S3 Study Protocol |
|  | Data sharing | 4 | Where and how the individual de-identified participant data (including data dictionary), statistical code and any other materials can be accessed | p.13 – Data availability section; Supporting Information – Supplementary File S1 (M9_Integrative_RCT_Dataset_v3.xlsx) |
|  | Funding and conflicts of interest | 5a | Sources of funding and other support (eg, supply of drugs), and role of funders in the design, conduct, analysis and reporting of the trial | p.2 – Abstract (“Funding: Unrestricted grant; funder had no role”); p.13 – Funding; Role of the funding source |
|  |  | 5b | Financial and other conflicts of interest of the manuscript authors | p.13 – Declaration of competing interest |
|  | **Introduction** | | |  |
|  | Background and rationale | 6 | Scientific background and rationale | pp.3–4 – Introduction (background on HPA/SNS, diurnal cortisol, hair cortisol, HRV) |
|  | Objectives | 7 | Specific objectives related to benefits and harms | p.4 – Rationale and objective; Hypotheses |
|  | **Methods** | | |  |
|  | Patient and public involvement | 8 | Details of patient or public involvement in the design, conduct and reporting of the trial | N/A – no formal patient or public involvement in design, conduct, or reporting |
|  | Trial design | 9 | Description of trial design including type of trial (eg, parallel group, crossover), allocation ratio, and framework (eg, superiority, equivalence, non-inferiority, exploratory) | p.1 – Design line “Parallel‑group, superiority, randomized, active‑controlled trial (allocation ratio 1:1)”; pp.4–5 – Methods: Study Design and Oversight |
|  | Changes to trial protocol | 10 | Important changes to the trial after it commenced including any outcomes or analyses that were not prespecified, with reason | No important changes after trial commencement; all prespecified outcomes and analyses reported (N/A) |
|  | Trial setting | 11 | Settings (eg, community, hospital) and locations (eg, countries, sites) where the trial was conducted | pp.4–5 – Methods: Study Design and Oversight (“Recruitment occurred 9 Mar 2025–30 Mar 2025 at the Center for New Medical Technologies, Novosibirsk, Russian Federation”); p.7 – Results: Participants and setting |
|  | Eligibility criteria | 12a | Eligibility criteria for participants | p.4 – Methods: Participants (inclusion and exclusion criteria) |
|  |  | 12b | If applicable, eligibility criteria for sites and for individuals delivering the interventions (eg, surgeons, physiotherapists) | pp.4–5 – Methods: Study Design and Oversight (single‑site trial at Center for New Medical Technologies); pp.5–6 – Methods: Interventions (therapists – clinical psychologists trained in the registered method, ≥2 per group) |
|  | Intervention and comparator | 13 | Intervention and comparator with sufficient details to allow replication. If relevant, where additional materials describing the intervention and comparator (eg, intervention manual) can be accessed | pp.5–6 – Methods: Interventions (INT and CTRL content, session length, group size, components, homework, fidelity/differentiation procedures); p.9 – Results: Exposure, Fidelity, and Adherence; Supplement Table S3 |
|  | Outcomes | 14 | Prespecified primary and secondary outcomes, including the specific measurement variable (eg, systolic blood pressure), analysis metric (eg, change from baseline, final value, time to event), method of aggregation (eg, median, proportion), and time point for each outcome | pp.5–6 – Methods: Outcomes (primary AUCg cortisol; secondaries: CAR, diurnal slope, sAA, cortisone, HCC, mechanistic targets); Salivary Sampling and Preanalytic Controls; Hair Cortisol Procedures; TSST Substudy; Process, safety, blinding, and assay QC |
|  | Harms | 15 | How harms were defined and assessed (eg, systematically, non-systematically) | p.6 – Methods: Process Measures, Expectancy/Masking, and Safety (NEQ, AE monitoring, CONSORT‑Harms); p.9 – Results: Safety, Blinding, and Assay Quality Control; Supplement Table S4 |
|  | Sample size | 16a | How sample size was determined, including all assumptions supporting the sample size calculation | p.6 – Methods: Sample Size and Power (assumptions, target N≈60–80, clustering/ICC, attrition allowance); protocol |
|  |  | 16b | Explanation of any interim analyses and stopping guidelines | N/A – no interim analyses or formal stopping guidelines were planned |
|  | Randomisation: |  |  |  |
|  | Sequence generation | 17a | Who generated the random allocation sequence and the method used | p.4 – Methods: Randomization and Masking (“computer‑generated, block‑stratified sequences …” by independent statistician) |
|  |  | 17b | Type of randomisation and details of any restriction (eg, stratification, blocking and block size) | p.4 – Methods: Randomization and Masking (1:1 allocation; block randomisation with stratification by sex, baseline symptom severity, and oral contraceptive status in women) |
|  |  |  |  | **Reported on page no.** |
|  | Allocation concealment mechanism | 18 | Mechanism used to implement the random allocation sequence (eg, central computer/telephone; sequentially numbered, opaque, sealed containers), describing any steps to conceal the sequence until interventions were assigned | p.4 – Methods: Randomization and Masking (“Allocation was concealed via a centralized assignment system”) |
|  | Implementation | 19 | Whether the personnel who enrolled and those who assigned participants to the interventions had access to the random allocation sequence | p.4 – Methods: Randomization and Masking (participants enrolled at the Center; independent statistician generated sequence; allocation via centralized system, investigators and assessors had no access to sequence) |
|  | Blinding | 20a | Who was blinded after assignment to interventions (eg, participants, care providers, outcome assessors, data analysts) | pp.4–5 – Methods: Randomization and Masking (assessors and laboratory personnel blinded; participants not explicitly informed of allocation); p.6 – Methods: Process Measures, Expectancy/Masking, and Safety (masking check at T1); p.9 – Results: Safety, Blinding, and Assay Quality Control |
|  |  | 20b | If blinded, how blinding was achieved and description of the similarity of interventions | pp.5–6 – Methods: Interventions (both arms as group‑based stress‑management programs of equal duration and contact); pp.4–5 – Methods: Randomization and Masking (participants not told which program was experimental; group‑guess at T1) |
|  | Statistical methods | 21a | Statistical methods used to compare groups for primary and secondary outcomes, including harms | p.6 – Methods: Statistical Analysis (ITT linear mixed‑effects models, covariates, FDR, sensitivity checks; harms analysis); pp.7–9 – Results (primary and secondary outcomes) |
|  |  | 21b | Definition of who is included in each analysis (eg, all randomised participants), and in which group | p.6 – Methods: Statistical Analysis (“primary intention‑to‑treat analysis” of all randomized participants); p.7 – Results: Participants and setting; Tables 1–5; Supplement Tables S1–S2 |
|  |  | 21c | How missing data were handled in the analysis | p.6 – Methods: Statistical Analysis (“Missing data were handled under ITT by maximum likelihood within the mixed‑model framework; per‑protocol and CACE sensitivity analyses and multiple imputation as robustness checks”) |
|  |  | 21d | Methods for any additional analyses (eg, subgroup and sensitivity analyses), distinguishing prespecified from post hoc | p.6 – Methods: Statistical Analysis (cluster‑robust SE, bootstrap, FDR families; TSST substudy); pp.8–9 – Results: ITT mixed‑effects models; TSST Substudy; Supplement Tables S1–S2 |
|  | **Results** | | |  |
|  | Participant flow, including flow diagram | 22a | For each group, the numbers of participants who were randomly assigned, received intended intervention, and were analysed for the primary outcome | p.7 – Results: Participants and setting (INT n=30; CTRL n=30); p.16 – Figure 1 (CONSORT participant flow diagram) |
|  |  | 22b | For each group, losses and exclusions after randomisation, together with reasons | p.16 – Figure 1 (no loss to follow‑up and no discontinuations; all 60 included in primary analysis) |
|  | Recruitment | 23a | Dates defining the periods of recruitment and follow-up for outcomes of benefits and harms | pp.4–5 – Methods: Study Design and Oversight (“Recruitment occurred 9 Mar 2025–30 Mar 2025 … T0, T1 (~8 weeks), T2 (~3 months), T3 (6‑month HCC)”); pp.5–6 – Methods: Outcomes and Hair Cortisol Procedures |
|  |  | 23b | If relevant, why the trial ended or was stopped | N/A – trial ran to completion as planned; not stopped early |
|  | Intervention and comparator delivery | 24a | Intervention and comparator as they were actually administered (eg, where appropriate, who delivered the intervention/comparator, how participants adhered, whether they were delivered as intended (fidelity)) | pp.5–6 – Methods: Interventions; Process Measures, Expectancy/Masking, and Safety (attendance, home practice, coder‑rated fidelity); p.9 – Results: Exposure, Fidelity, and Adherence; Supplement Table S3 |
|  |  | 24b | Concomitant care received during the trial for each group | N/A – participants continued usual care; concomitant treatments were not protocolised (baseline medications and changes recorded in eCRFs) |
|  | Baseline data | 25 | A table showing baseline demographic and clinical characteristics for each group | p.7 – Results: Participants and setting; p.17 – Table 1 (Baseline Characteristics of the Participants at Enrollment) |
|  | Numbers analysed,  outcomes and estimation | 26 | For each primary and secondary outcome, by group:  ● the number of participants included in the analysis  ● the number of participants with available data at the outcome time point  ● result for each group, and the estimated effect size and its precision (such as 95% confidence interval)  ● for binary outcomes, presentation of both absolute and relative effect size | pp.7–9 – Results: Primary Outcome; Key Secondary Salivary Outcomes; Long‑Term Cortisol Load; Mechanistic Target Engagement; TSST Substudy; pp.18, 20–22 – Tables 2–5; Supplement Tables S1–S2 |
|  | Harms | 27 | All harms or unintended events in each group | p.9 – Results: Safety, Blinding, and Assay Quality Control; Supplement Table S4 (adverse events, NEQ scores, assay CVs) |
|  | Ancillary analyses | 28 | Any other analyses performed, including subgroup and sensitivity analyses, distinguishing pre-specified from post hoc | p.6 – Methods: Statistical Analysis (sensitivity analyses; cluster‑robust SE, bootstrap; TSST substudy); p.9 – Results: ITT mixed‑effects models; TSST Substudy; Supplement Tables S1–S2 |
|  | **Discussion** | | |  |
|  | Interpretation | 29 | Interpretation consistent with results, balancing benefits and harms, and considering other relevant evidence | pp.10–12 – Discussion: Principal findings; Interpretation in the context of prior literature; Mechanistic considerations; Clinical and translational relevance |
|  | Limitations | 30 | Trial limitations, addressing sources of potential bias, imprecision, generalisability, and, if relevant, multiplicity of analyses | pp.11–12 – Discussion: Strengths and limitations; Generalisability; notes on multiplicity/FDR |

Citation: Hopewell S, Chan AW, Collins GS, Hróbjartsson A, Moher D, Schulz KF, et al. CONSORT 2025 Statement: updated guideline for reporting randomised trials. BMJ. 2025; 388:e081123. <https://dx.doi.org/10.1136/bmj-2024-081123>
© 2025 Hopewell et al. This is an Open Access article distributed under the terms of the Creative Commons Attribution License (<https://creativecommons.org/licenses/by/4.0/>), which permits unrestricted use, distribution, and reproduction in any medium, provided the original work is properly cited.

*We strongly recommend reading this statement in conjunction with the CONSORT 2025 Explanation and Elaboration and/or the CONSORT 2025 Expanded Checklist for important clarifications on all the items. We also recommend reading relevant CONSORT extensions. See [www.consort-spirit.org](http://www.consort-spirit.org).
