# Supplementary material for: Integrative group psychotherapy reduces daily cortisol output and hair cortisol: A randomized active‑controlled trial with multi‑day profiling
Source: PLoS One. 2026 Jul 23;21(7):e0352095. doi: 10.1371/journal.pone.0352095 (PMC13395371; doi:10.1371/journal.pone.0352095)
Supplement: S2 File — (PDF) [file pone.0352095.s011.pdf]

ПРОТОКОЛ КЛИНИЧЕСКОГО ИССЛЕДОВАНИЯ

Название исследования (рус.):
Интегративная групповая психотерапия снижает суточную продукцию кортизола и уровень кортизола в волосах: рандомизированное исследование с активным контролем и многодневным профилированием

Название исследования (англ.):
Integrative group psychotherapy reduces daily cortisol output and hair cortisol: A randomized active-controlled trial with multi-day profiling

Код протокола: M9

Версия протокола: 1.2
Дата версии: 10 декабря 2024 г.

Регистрация клинического исследования:
ClinicalTrials.gov, идентификатор NCT06863948
Дата регистрации: 07.03.2025 (до включения первого участника).

Инициатор/спонсор:
Центр новых медицинских технологий, г. Новосибирск, Российская Федерация
при финансовой поддержке компании «Therapy of the Soul» (Новосибирск, РФ).

Главный исследователь:
д.м.н. Евгений Поку́шалов
Центр новых медицинских технологий
630090, Россия, г. Новосибирск
E-mail: E.Pokushalov@gmail.com

Ответственный за проведение психотерапевтического вмешательства:
Евгений Теребенин (разработчик интегративного метода; тренер терапевтов).

1. КРАТКАЯ СУТЬ ПРОТОКОЛА

- **Дизайн:** параллельное, рандомизированное, контролируемое клиническое исследование (superiority-дизайн), две группы лечения, отношение рандомизации 1:1.
- **Популяция:** взрослые мужчины и женщины 18–60 лет с субклиническими–умеренными симптомами тревоги/депрессии и хроническим психосоциальным стрессом, без тяжелой психиатрической патологии.
- **Интервенции:**
  - *INT* — интегративная групповая психотерапия (8 еженедельных сессий, 2–3 часа; социально-аффективные навыки, медленное дыхание с HRV-биологической обратной связью, когнитивная переоценка/стресс-майндсет + осознанность/интероцепция).

- *CTRL* — активный контроль: дозо- и формат-сопоставимая групповая программа с акцентом на психообразование и общие навыки стресс-менеджмента, без специфических активных компонентов INT.

- **Основная конечная точка:** изменение суточной продукции кортизола по данным трехдневного слюнного профиля (AUCg, nmol/L·h) от исходного визита (T0) до конца лечения (~8 нед., T1).

- **Ключевые вторичные конечные точки:**
  - кортизоловый ответ пробуждения (CAR, AUCi);
  - диурнальный наклон секреции кортизола;
  - AUCg слюнного кортизона;
  - AUCg слюнной α-амилазы (sAA);
  - концентрация кортизола в волосах (HCC, T0, T1 и 6-месячный визит T3);
  - показатели вариабельности сердечного ритма (HRV) и частоты дыхания;
  - психометрические шкалы (ERQ-Reappraisal, Self-Compassion Scale, MAIA-2 и др.);
  - реактивность кортизола на Trier Social Stress Test (TSST) в подвыборке.

- **Объем выборки:** минимум 60 рандомизированных участников (30 в каждой группе).

- **Продолжительность участия одного участника:** около 6 месяцев (T0→T3).

- **Место проведения:** Центр новых медицинских технологий (Новосибирск, РФ).

2. ОБОСНОВАНИЕ ИССЛЕДОВАНИЯ

2.1. Научный фон

Хронический психосоциальный стресс ассоциирован с дисрегуляцией гипоталамо-гипофизарно-надпочечниковой (ГГН) оси, повышенным риском аффективных и соматических заболеваний и неблагоприятными исходами здоровья. Суточный профиль кортизола (общая площадь под кривой AUCg, ответ пробуждения CAR, диурнальный наклон) является информативным маркером нагрузки на ГГН-ось. Дополнительно концентрация кортизола в волосах отражает накопленную экспозицию глюкокортикоидам на протяжении недель и месяцев.

Существующие поведенческие и психотерапевтические вмешательства (майндфулнес, когнитивно-поведенческая терапия и др.) демонстрируют неоднородные эффекты на показатели кортизола, во многом из-за методологических различий (однодневные измерения, отсутствие контроля качества, отсутствие активного контроля и т.п.).

2.2. Интегративная групповая психотерапия

Разработан интегративный групповой протокол, сочетающий:

1. **Социально-аффективные навыки** (эмпатия, сострадание, поддерживающая коммуникация);

2. **Автономную регуляцию** через медленное диафрагмальное дыхание (~6 вдохов/мин) с биологической обратной связью по вариабельности сердечного ритма (HRV-biofeedback);

3. **Когнитивную переоценку и работу со стресс-майндсетом**, встроенные в практики осознанности и интероцептивную фокусировку.

Предполагается, что данный комплекс воздействует на нейрогуморальные механизмы стресса (ГГН-ось и симпатическую нервную систему), а также на когнитивно-аффективные механизмы регуляции.

2.3. Цель исследования

Оценить, превосходит ли интегративная групповая психотерапия активный контроль по влиянию на суточную продукцию кортизола и связанные с ней физиологические и психометрические показатели у взрослых с субклиническими–умеренными симптомами тревоги/депрессии.

2.4. Гипотезы

1. По сравнению с активным контролем, интегративная терапия приведет к более выраженному снижению:
   - суточного AUCg слюнного кортизола;
   - концентрации кортизола в волосах (HCC).
2. Интервенция окажет благоприятное влияние на CAR и диурналъный наклон кортизола, а также на AUCg слюнного кортизона и sAA.
3. В подвыборке участников интегративная терапия снизит пиковый ответ кортизола на TSST.
4. Изменения физиологических показателей будут опосредованы улучшением проксимальных мишеней: HRV, частоты дыхания, навыков когнитивной переоценки, самосострадания и интероцептивной осознанности.

3. ДИЗАЙН ИССЛЕДОВАНИЯ

3.1. Тип исследования

- Интервенционное, одноцентровое, рандомизированное, параллельное, актив-контролируемое клиническое исследование.
- Отношение рандомизации 1:1 (INT:CTRL).

- Дизайн «superiority» — проверка превосходства интегративной терапии над активным контролем.

3.2. Схема визитов и временные точки

- **T0 (исходный визит, неделя 0):** скрининг критериев включения/исключения, информированное согласие, сбор базовых данных, инструктаж по сбору слюны и волос, проведение измерений HRV, психометрических шкал; старт трехдневного слюнного профиля и, при согласии, TSST-подисследования.

- **T1 (конец лечения, ~8 недель):** повторное проведение всех процедур, аналогичных T0 (без повторного скрининга), сбор трехдневного слюнного профиля, HRV, психометрических шкал, забор волос.

- **T2 (~3 месяца после T0):** повторный трехдневный слюнный профиль, HRV, психометрические шкалы (без вмешательства между T1 и T2).

- **T3 (6 месяцев после T0):** забор волос для HCC, сбор информации о последующих вмешательствах/изменениях образа жизни.

Полный период участия одного участника — до 6 месяцев.

3.3. Продолжительность исследования

- Планируемое начало набора: после утверждения протокола и регистрации на ClinicalTrials.gov.

- Набор продолжается до достижения требуемого количества рандомизированных участников (минимум 60).

- Ожидаемая общая продолжительность исследования (от первого включенного участника до последнего визита последнего участника) — около 9–12 месяцев.

4. УЧАСТНИКИ

4.1. Критерии включения

1. Мужчины и женщины в возрасте 18–60 лет.

2. Проживание в г. Новосибирске или в доступности для регулярного посещения групповых сессий.

3. Наличие субклинических или умеренных симптомов тревоги/депрессии (по шкалам STAI-Trait, BDI-II — уровни, требующие психотерапии, но не неотложного психиатрического вмешательства).

4. Готовность соблюдать протокол: посещать 8 групповых сессий, выполнять домашние задания, собирать слюну и образцы волос по инструкции.

5. Подписанное **информированное добровольное согласие** на участие в исследовании, включая согласие на обработку персональных данных и использование обезличенных данных в научных целях.

4.2. Критерии исключения

1. Тяжелые психические расстройства (шизофрения, биполярное расстройство, тяжелые эпизоды большой депрессии с высоким суицидальным риском, активная зависимость от психоактивных веществ и др.), требующие специализированной психиатрической помощи.
2. Выраженные когнитивные нарушения, препятствующие пониманию сути вмешательства и заполнению анкет.
3. Ночная сменная работа или нерегулярный график сна (менее 5 ночей сна в привычное время в неделю), значительно нарушающий циркадный ритм.
4. Диагностированные тяжелые нарушения сна (например, тяжелая обструктивная апноэ сна, нелеченная) без адекватной терапии.
5. Эндокринные заболевания, существенно влияющие на кортизол (синдром Кушинга, первичная/вторичная надпочечниковая недостаточность и т.п.).
6. Прием системных глюкокортикоидов или других лекарственных средств, существенно влияющих на ГГН-ось, в течение последних 3 месяцев.
7. Текущая психотерапия аналогичной направленности (интенсивная групповая или индивидуальная терапии с сильным перекрытием по техникам); участие в других клинических исследованиях.
8. Беременность или планируемая беременность в ближайшие 6 месяцев.
9. Любые другие состояния, которые по мнению исследователя делают участие пациента нежелательным или небезопасным.

Для женщин фиксируются статус приёма комбинированных оральных контрацептивов и фаза менструального цикла; эти параметры используются при стратификации и анализе.

4.3. Рекрутирование и скрининг

- Реклама исследования (объявления, соцсети, сайт Центра и др.) приглашает потенциальных участников с указанием общих критериев.
- На первичном контакте по телефону/онлайн проводится предварительный скрининг.
- Подходящие лица приглашаются на очную встречу в Центр, где проводится подробный скрининг, объяснение сути исследования и выдача информационного листка.
- При согласии участник подписывает форму информированного добровольного согласия.

4.4. Информированное согласие

- Форма информированного согласия включает описание цели исследования, процедур, рисков и ожидаемой пользы, альтернатив, добровольности участия и возможности отмены участия без ущерба для медицинской помощи.
- Участник получает достаточное время для принятия решения; допускается присутствие близкого человека.
- Исследователь отвечает на вопросы, после чего участник подписывает две копии формы (одна — участнику, другая — в архив исследования).

5. ИНТЕРВЕНЦИИ

5.1. Общие положения

- Оба вмешательства проводятся в группах по 8–12 человек.
- Всего 8 еженедельных сессий по 2–3 часа.
- Каждую группу ведут два подготовленных терапевта-психолога.
- Для обеих групп используются стандартизированные сценарии сессий и формы оценки соблюдения протокола (fidelity/differentiation).

5.2. Интегративная групповая психотерапия (INT)

Цель: обучение навыкам социально-аффективной поддержки, автономной регуляции и когнитивной переоценки для снижения хронической стрессовой нагрузки.

Структура типичной сессии:

1. *Краткий чек-ин* (10–20 мин): круг, обсуждение самочувствия, опыта выполнения домашних заданий.
2. *Психообразование и ввод новой темы* (30–40 мин): краткий теоретический блок с обсуждением.
3. *Практические упражнения* (40–60 мин): работа в парах/триадах и малых группах.
4. *Практика медленного дыхания с HRV-биофидбеком* (10–20 мин).
5. *Заключительный круг и назначение домашнего задания* (15–20 мин).

Компонент 1 – социально-аффективные навыки

- Отработка эмпатического слушания, подкрепляющей обратной связи, выражения поддержки.
- Упражнения в парах («поддерживающий слушатель», «трудный разговор»), короткие практики сострадания и благодарности.
- Домашние задания: минимум 3 эпизода целенаправленного применения навыков поддержки в течение недели с краткой записью (дневник).

Компонент 2 – автономная регуляция (медленное дыхание и HRV-биофидбек)

- Обучение диафрагмальному дыханию с частотой примерно 6 вдохов в минуту (0,1 Гц).
- Использование датчика пульса (палец/ухо) и компьютерного/мобильного приложения HRV-биофидбека для визуализации вариабельности сердечного ритма.
- Групповая практика 10–15 минут/сессия с целью достижения «резонансного» рисунка HRV.
- Домашнее задание: не менее 20 минут в день (с устройством или по аудиозаписи).

Компонент 3 – когнитивная переоценка и стресс-майндсет

- Обучение распознаванию автоматических стрессовых мыслей, замене их альтернативными интерпретациями.
- Написание кратких ABC-форм, ролевые игры в ситуациях социальной оценки (публичное выступление, конфликт и др.).
- Обсуждение beliefs о стрессе («стресс как разрушающий» vs «стресс как ресурс»).
- Домашние задания: минимум 3 эпизода письменной переоценки стрессовых ситуаций в неделю.

Осознанность и интероцепция

- Встраивание коротких практик осознанного дыхания, сканирования тела, фокусировки на ощущениях сердца/груди в каждую сессию.
- Цель — улучшение интероцептивной осознанности (MAIA-2).

Терапевты и супервизия

- Терапевты — клинические психологи/психотерапевты, прошедшие не менее 40 часов обучения интегративному методу (лектории, отработка навыков, наблюдение за сессиями).
- Еженедельная групповая супервизия под руководством Е. Теребенина.
- Все сессии аудиозаписываются для целей контроля качества.

Контроль соблюдения протокола (fidelity/differentiation)

- Два независимых, ослепленных к гипотезам исследования кодера прослушивают выборочные аудиозаписи и заполняют стандартизированные формы:
  - количество минут, отведенных на каждый компонент (социально-аффективный, дыхание/HRV, переоценка, майндфулнес, психообразование);
  - оценка соблюдения протокола (0–6);
  - наличие «запрещенного» контента (контаминация).

5.3. Активный контроль (CTRL)

Цель: предоставить участникам поддержку и общие знания о стрессе и здоровье при исключении специфических активных компонентов INT.

Основные характеристики:

- 8 групповых сессий, 2–3 часа, те же терапевты (обучены и в этом протоколе).
- Основной упор на психообразование и обсуждение: биология стресса, влияние сна, питания, физической активности, социальной поддержки; общие техники релаксации и тайм-менеджмента.
- Не включаются: структурированная практика медленного дыхания/HRV-биофидбека, целенаправленная тренировка сострадания в парах, специфичные упражнения на переоценку под социальной оценкой.

Домашние задания: чтение раздаточных материалов, ведение дневника стресса, применение общих рекомендаций по образу жизни.

Фиделити и дифференциация оцениваются теми же формами; ожидается, что по минутам на компоненты профили INT и CTRL существенно различаются (что и будет проверено).

6. КОНЕЧНЫЕ ТОЧКИ

6.1. Первичная конечная точка

- **Изменение среднесуточной продукции слюнного кортизола (AUCg, nmol/L·h)**, усредненной по трем последовательным дням на каждом визите (T0 и T1).
    - Расчет по формуле Пруесснера (AUCg).
    - Основной анализ: разница изменений (ΔT1–T0) между группами INT и CTRL (ITT-анализ) и оценка эффекта во времени в смешанной модели.

6.2. Ключевые вторичные физиологические конечные точки

1. **CAR (cortisol awakening response):**
    - AUCi (nmol/L·h) по трем точкам (0, +30, +45 мин после пробуждения) на каждом из трех дней.
2. **Диурнальный наклон кортизола:**
    - линейный наклон (nmol/L/час) между утренними и вечерними точками.
3. **AUCg слюнного кортизона (nmol/L·h)**.
4. **AUCg слюнной α-амилазы (sAA, U/mL·h)** и диурнальный наклон sAA.

5. **Концентрация кортизола в волосах (HCC, pg/mg)** на T0, T1 и T3 (1-см проксимальный сегмент волос).

6.3. Механистические (проксимальные) конечные точки

1. Показатели HRV в покое (RMSSD, HFnu).
2. Частота дыхания (вдохов/мин).
3. Шкала ERQ – субшкала «переоценка» (1–7).
4. Шкала самосострадания SCS (1–5).
5. Шкала интероцептивной осознанности MAIA-2 (0–5).

6.4. Клинические и психологические исходы

- Баллы по шкалам депрессии (BDI-II), тревоги (STAI-Trait/State), субъективного стресса и качества жизни.

6.5. TSST-подисследование

- Пиковая концентрация слюнного кортизола (nmol/L) во время TSST до и после вмешательства (пред- и пост-интервенционный TSST).
- Изменение (post–pre) и междугрупповое сравнение Δ.

6.6. Исходы безопасности и переносимости

- Частота и характер нежелательных явлений (НЯ) и серьезных нежелательных явлений (СНЯ).
- Негативные эффекты психотерапии по опроснику NEQ.

7. МЕТОДЫ СБОРА ДАННЫХ

7.1. Сбор слюны

- На каждом слюнном блоке участник собирает слюну в течение 3 последовательных дней.
- В каждый день — 5 проб:
    1. При пробуждении (0 мин);
    2. Через 30 мин после пробуждения;
    3. Через 45 мин после пробуждения;
    4. Дневная проба (~6–8 часов после пробуждения);

5. Вечерняя проба (перед сном).

- Участникам выдаются:
   1. наборы пробирок,
   2. подробные письменные инструкции,
   3. напоминания по SMS/мессенджеру.
- Соблюдение времени документируется:
   1. запись времени на этикетке,
   2. при наличии — электронная отметка,
   3. актиметрия для подтверждения времени пробуждения.
- Перед забором: 30 мин без еды/напитков/курения, 10 мин без чистки зубов.

7.2. Лабораторный анализ слюны

- Аналиты: кортизол, кортизон, α-амилаза.
- Метод анализа — валидация согласно стандартам лаборатории (иммуноанализ или LC-MS; в протоколе допускается любой валидированный метод, окончательный метод прописывается в SOP лаборатории).
- Отслеживаются: пределы обнаружения/квантификации (LoD/LoQ), внутрисерийные и межсерийные коэффициенты вариации, номера планшетов и серий, количество циклов заморозки-разморозки.

7.3. Сбор волос

- Срез 1-см проксимального сегмента волос с затылочной области (несколько прядей), максимально близко к коже.
- Образец маркируется и хранится при комнатной температуре до анализа.
- Анализ HCC — в специализированной лаборатории, валидация методики проводится согласно международным рекомендациям.

7.4. Измерение HRV и частоты дыхания

- Регистрация ЭКГ (или фотоплетизмограмма) в положении сидя/полулежа, в спокойном состоянии, не менее 5 мин.
- Анализ HRV (RMSSD, HFnu) по стандартным алгоритмам.
- Частота дыхания либо по респираторному датчику, либо по анализу вариабельности R–R с учетом дыхательной модуляции.

7.5. Психометрические шкалы

- STAI, BDI-II, ERQ, SCS, MAIA-2 и др. заполняются участниками в бумажном или электронном виде на T0, T1, T2 (часть шкал — только T0/T1).

7.6. TSST

- Стандартный протокол: подготовка 5 мин, публичное выступление 5 мин, устный счет 5 мин перед экспертом(ами).
- Сбор слюны: базовая проба, несколько проб во время и после теста (конкретное расписание описано во внутреннем SOP).
- Проводится до и после курса терапии (T0 и T1) у подвыборки участников, давших отдельное согласие.

8. РАНДОМИЗАЦИЯ, СКРЫТИЕ РАСПРЕДЕЛЕНИЯ И МАСКИРОВАНИЕ

8.1. Генерация последовательности рандомизации

- Используется компьютер-генерируемая случайная последовательность с блочной стратификацией.
- Стратификационные факторы:
  - пол (мужчина/женщина);
  - исходная выраженность симптомов (по BDI-II/STAI, дихотомизация по медиане);
  - для женщин — прием комбинированных оральных контрацептивов (да/нет).

8.2. Сокрытие распределения

- Последовательность рандомизации формируется независимым статистиком до начала набора.
- Назначение группы осуществляется централизованно (телефон/электронная система) после завершения всех базовых измерений T0 и подтверждения критериев включения/исключения.
- Лица, оценивающие исходы и выполняющие лабораторные анализы, **не имеют доступа** к последовательности рандомизации.

8.3. Маскирование

- Участники не информируются о гипотезе «какая группа является активной» и о конкретных различиях между программами; обе описываются как «эффективные методы управления стрессом».
- Оценщики исходов и лабораторный персонал ослеплены к группе.

- В конце курса (T1) участникам предлагается угадать, в какой группе они были, с оценкой уверенности — для оценки успешности маскирования.

9. РАЗМЕР ВЫБОРКИ И ОБОСНОВАНИЕ

Расчет мощности основан на первичной конечной точке — изменении AUCg слюнного кортизола T0→T1.

- Предыдущие исследования с подобными вмешательствами и мультидневными измерениями показали эффекты порядка d ≈ 0,6–0,8 по изменению AUCg.
- При $\alpha = 0{,}05$ (двусторонний критерий) и мощности 80 % обнаружение разницы $\Delta \approx 0{,}6$ SD между группами при использовании смешанных моделей (учет повторных измерений и умеренной внутрисубъектной корреляции ~0,5) достигается при N≈25–30 человек на группу.
- Учитывая возможное выбывание до 15–20 %, планируемый минимум — **60 рандомизированных участников (30 в каждой группе)**.

При наличии ресурсов допускается увеличение выборки до 80 участников, что повысит мощность анализов подгрупп и TSST-подисследования.

10. СТАТИСТИЧЕСКИЙ АНАЛИЗ

10.1. Общие принципы

- Основной анализ — по принципу **intention-to-treat (ITT)**: все рандомизированные участники анализируются в тех группах, к которым они были рандомизированы, независимо от фактического объема вмешательства.
- Дополнительные анализы: **per-protocol** и анализ complier average causal effect (CACE), ограниченный участниками с адекватным посещением и выполнением домашней работы.

10.2. Модели для основных физиологических исходов

- Используются **линейные смешанные модели (LMM)** с фиксированными эффектами времени (T0, T1, T2), группы (INT/CTRL) и их взаимодействия (time×group), а также случайными перехватами для участника и для терапевтического кластера (терапевт, вложенный в группу).
- Для гормональных показателей применяются лог-преобразования; результаты интерпретируются как процентные изменения (ratio of change).
- Ковариаты: время пробуждения, соблюдение временных окон при сборе слюны, показатели курения/кофеина, лабораторные параметры (планшет/серия, число заморозка-разморозка), для женщин — статус ОК и фаза цикла.

10.3. Коррекция множественных сравнений

- Для заранее заданных семейств вторичных исходов (CAR/наклон, sAA, кортизон, HCC, механистические показатели) применяется контроль **ложного открытия Benjamini–Hochberg (FDR, q=0,05)**.

10.4. Обращение с пропущенными данными

- В смешанных моделях пропуски исходных переменных обрабатываются методом максимального правдоподобия при предположении MAR.
- В чувствительных анализах возможно использование множественной иммутации.

10.5. Анализ TSST-подисследования

- Для пикового кортизола в TSST используется LMM с факторами фаза (pre/post), группа и их взаимодействие.
- Основной интерес представляет interaction term (post×INT).

10.6. Анализ безопасности и процессных показателей

- Частоты НЯ и СНЯ сравниваются между группами с использованием точного критерия Фишера.
- Показатели посещаемости, выполненности домашнего задания и фиделити описываются описательной статистикой; межгрупповые сравнения — t-тестами/непараметрическими критериями.

10.7. Программное обеспечение

- Анализ проводится с использованием стандартных статистических пакетов (R, Stata, SPSS или эквивалент).
- Все скрипты анализа сохраняются и при необходимости могут быть предоставлены.

11. УПРАВЛЕНИЕ ДАННЫМИ И КОНФИДЕНЦИАЛЬНОСТЬ

- Для каждого участника создается уникальный код; персональные данные хранятся отдельно от исследовательской базы.
- Доступ к реидентифицирующей информации имеют только главный исследователь и уполномоченные сотрудники Центра.
- Электронные данные защищены паролями и резервируются на защищенных серверах.
- Хранение данных — не менее 5 лет после публикации результатов или согласно требованиям локальных нормативных актов.

12. МОНИТОРИНГ БЕЗОПАСНОСТИ

- Все НЯ и СНЯ фиксируются исследователем, включая дату, тяжесть, причинно-следственную связь с вмешательством и исход.
- О серьезных нежелательных явлениях сообщается в Этический комитет в соответствии с локальными требованиями.
- При возникновении угрозы безопасности участника исследователь может временно приостановить или прекратить участие конкретного участника либо исследование в целом.

13. ЭТИЧЕСКИЕ АСПЕКТЫ

- Исследование проводится в соответствии с Хельсинкской декларацией, правилами GCP и законодательством РФ.
- Протокол, форма информированного согласия и сопутствующие материалы утверждены **Этическим комитетом Центра новых медицинских технологий** (решение № 0182CS_2024 от 10.12.2024 г.).
- Участие добровольное; участник может выйти из исследования в любое время без объяснения причин и без ущерба для последующей медицинской помощи.

14. РАСПРОСТРАНЕНИЕ РЕЗУЛЬТАТОВ

- Результаты исследования будут представлены в виде научных публикаций и докладов на конференциях.
- Публикуются только обобщенные обезличенные данные; идентификация конкретных участников исключена.
- Основная публикация планируется в международном журнале с рецензированием; дополнительные анализы и открытые данные будут доступны в виде вспомогательных материалов и/или в репозитории открытой науки.

15. РОЛЬ СПОНСОРА И КОНФЛИКТ ИНТЕРЕСОВ

- Компания «Therapy of the Soul» обеспечивает безвозмездное финансирование, необходимое для проведения групповых занятий и технической поддержки исследования.
- Спонсор **не участвует** в разработке дизайна, сборе и анализе данных, интерпретации результатов и подготовке публикаций.

- Евгений Теребенин является разработчиком и владельцем зарегистрированного метода интегративной психотерапии; его роль в исследовании ограничивается обучением терапевтов и супервизией проведения сессий в рамках протокола. Он не участвует в анализе данных и интерпретации результатов.

Конец протокола (версия 1.2 от 10.12.2024 г.).
