# Supplementary material for: Integrative group psychotherapy reduces daily cortisol output and hair cortisol: A randomized active‑controlled trial with multi‑day profiling
Source: PLoS One. 2026 Jul 23;21(7):e0352095. doi: 10.1371/journal.pone.0352095 (PMC13395371; doi:10.1371/journal.pone.0352095)
Supplement: S3 File — (PDF) [file pone.0352095.s012.pdf]

CLINICAL TRIAL PROTOCOL

Study title (Russian original):
"Интегративная групповая психотерапия снижает суточную продукцию кортизола и уровень кортизола в волосах: рандомизированное исследование с активным контролем и многодневным профилированием"

Study title (English):
Integrative group psychotherapy reduces daily cortisol output and hair cortisol: A randomized active-controlled trial with multi-day profiling

Protocol code: M9

Protocol version: 1.0
Version date: 10 December 2024

Study type:
Parallel-group, randomized, active-controlled superiority trial (allocation ratio 1:1).

Clinical trial registration:
ClinicalTrials.gov, identifier **NCT06863948**
Date of registration: 07 March 2025
First participant enrolled: 09 March 2025 (prospective registration).

1. Sponsor and study site

Sponsor / funding source
Therapy of the Soul (Novosibirsk, Russian Federation) – unrestricted grant to support trial conduct and delivery of the psychotherapeutic intervention.

Coordinating center
Center for New Medical Technologies
630090 Novosibirsk, Russian Federation

Principal Investigator (PI)
Evgeny Pokushalov, MD, PhD
Center for New Medical Technologies, Novosibirsk

Co-investigators
Clinical and research staff of the Center for New Medical Technologies and affiliated clinical psychologists/psychotherapists, as listed in the internal delegation log.

2. Scientific background and rationale

Chronic psychosocial stress is associated with dysregulation of the hypothalamic–pituitary–adrenal (HPA) axis and the sympathetic nervous system, which in turn increases the risk of both somatic and mental disorders. Dynamic characteristics of the diurnal cortisol profile—total daily output (AUCg), the cortisol awakening response (CAR), and the slope of decline from morning to bedtime

—are established biomarkers of stress-system load. Hair cortisol concentration (HCC) provides a complementary readout of long-term glucocorticoid exposure over weeks to months.

Few randomized trials of psychotherapeutic interventions combine:

- multi-day ambulatory salivary profiling of cortisol/cortisone and α-amylase;
- long-term HCC measures;
- an active control condition matched for contact time;
- mechanistic targets such as heart rate variability (HRV), breathing, and cognitive and socio-affective regulation.

An integrative group program developed by Evgeny Terebenin combines social-affective skills training, slow diaphragmatic breathing with HRV-biofeedback, and cognitive reappraisal/stress-mindset work embedded in mindfulness and interoceptive awareness practice. The program is expected to reduce daily glucocorticoid output and improve autonomic balance through coordinated changes in HPA and sympathetic function.

3. Objectives and hypotheses

3.1 Primary objective

To determine whether an integrative group psychotherapy program, compared with a dose-matched active control, reduces daily salivary cortisol output (3-day average AUCg) in adults with subclinical to moderate anxiety/depression symptoms.

3.2 Secondary objectives

1. To evaluate the effects of the intervention on:
    - cortisol awakening response (CAR, AUCi),
    - diurnal cortisol slope,
    - salivary cortisone AUCg,
    - salivary α-amylase (sAA) AUCg and diurnal slope.

2. To assess changes in hair cortisol concentration (HCC) at the end of treatment (~8 weeks) and at 6-month follow-up.
3. To assess intervention effects on proximal mechanistic targets: resting HRV, resting breathing rate, cognitive reappraisal, self-compassion, and interoceptive awareness.
4. In a TSST substudy, to examine changes in cortisol reactivity to standardized laboratory psychosocial stress.

5. To evaluate safety, tolerability, and negative effects of psychotherapy.

3.3 Hypotheses

1. Compared with active control, the integrative group psychotherapy will produce larger reductions in daily salivary cortisol AUCg and HCC.
2. The intervention will beneficially alter CAR, diurnal cortisol slope, and AUCg of cortisone and sAA.
3. The intervention will reduce TSST-evoked cortisol reactivity (exploratory hypothesis with limited power).
4. Changes in physiological outcomes will be mediated by improvements in HRV, breathing, and cognitive/socio-affective regulatory skills.

4. Study design

- Single-site, parallel-group, randomized, active-controlled superiority trial.
- Allocation ratio 1:1 (INT:CTRL).
- Intervention duration: 8 weeks (8 weekly group sessions).
- Assessment time points:
  - **T0** – baseline (pre-intervention),
  - **T1** – end of treatment (~8 weeks),
  - **T2** – post-treatment follow-up (~3 months from T0),
  - **T3** – 6-month follow-up for HCC only.

5. Study population

5.1 Inclusion criteria

1. Age 18–60 years.
2. Subclinical to moderate anxiety and/or depressive symptoms (BDI-II, STAI-Trait; specific cut-offs specified in the working SOP).
3. Willingness and ability to attend weekly group sessions for 8 weeks and complete homework.
4. Ability and willingness to comply with saliva and hair collection procedures and psychophysiological assessments.

5. Provision of written informed consent.

5.2 Exclusion criteria

1. Severe psychiatric disorder (e.g., psychotic disorder, bipolar I disorder, acute severe major depression with high suicide risk) requiring specialized care.
2. Current substance use disorder (other than nicotine).
3. Severe somatic illness that may affect cortisol metabolism (e.g., Cushing's syndrome, severe hepatic or renal failure).
4. Systemic glucocorticoid therapy or other medications with major impact on the HPA axis in the last 3 months.
5. Shift work including night shifts or pronounced sleep-wake disturbances.
6. Pregnancy or breastfeeding.
7. Concurrent participation in another interventional trial.
8. Any other condition judged by the investigator to make participation unsafe or infeasible.

For women, menstrual cycle phase and combined oral contraceptive (OC) use are recorded and considered at the design and analysis stages.

6. Recruitment and screening

Recruitment is carried out through:

- advertisements on the website and social media of the Center for New Medical Technologies;
- informational materials in clinics and universities;
- referrals from healthcare professionals.

Steps:

1. **Pre-screening** (phone/online): brief assessment of key inclusion/exclusion criteria.
2. **On-site screening visit** at the Center:
   - detailed history and clinical assessment;
   - completion of BDI-II, STAI, and other baseline questionnaires;
   - explanation of study objectives and procedures;
   - provision of the participant information sheet and informed consent form.

3. Eligible and willing individuals sign written informed consent and proceed to baseline assessments.

7. Randomization and masking

7.1 Sequence generation

Randomization is performed by an independent statistician using a computer-generated sequence with:

- allocation ratio 1:1;
- block randomization with variable block sizes;
- stratification by sex, baseline symptom severity, and OC use among women.

7.2 Allocation concealment

The allocation list is stored in a secure file accessible only to the randomization coordinator. After completion of baseline (T0) assessments and confirmation of eligibility, the coordinator assigns participants to INT or CTRL according to the concealed sequence.

7.3 Masking (blinding)

- **Blinded:** outcome assessors and laboratory personnel responsible for salivary and hair analyses are blinded to group assignment.
- **Participants:** are not explicitly told which program is considered “experimental” vs “control”; both are described as evidence-based stress-management group programs.
- At T1, participants are asked to guess their group assignment and rate confidence to assess the success of masking.

8. Interventions

8.1 Integrative group psychotherapy (INT)

Format

- 8 weekly group sessions, each 2–3 hours.
- Group size: 8–12 participants.
- Delivered by two trained clinical psychologists/psychotherapists.

Typical session structure

1. Brief check-in and review of home practice (~20 min).
2. Introduction of a core skill/topic (~30–40 min).
3. In-session practice (dyads/triads/small groups, ~40–60 min).
4. Slow diaphragmatic breathing with HRV-biofeedback (10–20 min).
5. Debrief, questions, and assignment of homework (~15–20 min).

Components

1. **Social-affective skills**
   - empathic listening, supportive communication, compassion;
   - structured partner dialogues, sharing exercises;
   - brief gratitude and compassion practices.
2. **Autonomic regulation (slow breathing + HRV-biofeedback)**
   - training in diaphragmatic breathing at ~6 breaths/min;
   - use of a pulse sensor and HRV-biofeedback software;
   - goal: resonance-frequency breathing with sinusoidal HRV pattern;
   - prescribed home practice ≥20 min/day using audio guides or devices.
3. **Cognitive reappraisal and stress mindset**
   - identification and restructuring of automatic stress-related thoughts (ABC forms);
   - role-plays of stressful social situations (e.g., conflict, evaluation);
   - discussion of stress mindsets (stress-as-enhancing vs stress-as-debilitating).

Mindfulness and interoceptive awareness practices (brief breathing, body scan, awareness of sensations in chest/heart) are interwoven throughout sessions.

The integrative group psychotherapy follows a registered proprietary method developed by **Evgeny Terebenin**. His role in the trial is limited to therapist training, supervision, and partial delivery of the intervention. He is not involved in trial design, data analysis, result interpretation, or publication decisions.

Homework

Participants keep a weekly practice diary recording minutes spent in each component (social-affective, breathing/HRV-biofeedback, reappraisal, mindfulness).

Fidelity and differentiation

- All sessions are audio-recorded.
- Two independent coders, blinded to hypotheses, complete standardized checklists for:
  - minutes per component;
  - adherence/competence (0–6);
  - presence/absence of "forbidden" content from the control arm.

8.2 Active control (CTRL)

Format

- Same structure: 8 group sessions, 2–3 hours each; 8–12 participants; same therapist qualifications.

Content

- Psychoeducation about stress, health, sleep, physical activity, and general coping strategies.
- Discussion-based groups and simple relaxation techniques.
- No structured resonance-frequency breathing or HRV-biofeedback;
- no systematic compassion dyads or targeted reappraisal practice under social-evaluation conditions.

Homework and monitoring

- Participants receive reading materials, self-monitoring tasks, and general stress-management exercises with a workload comparable to INT.
- Attendance, homework minutes, and fidelity/differentiation are assessed exactly as in INT to confirm separation of active ingredients.

9. Visit schedule and procedures

Screening visit (pre-randomization)

- Informed consent.
- Medical and psychiatric history.
- Baseline symptom questionnaires (BDI-II, STAI, etc.).
- Eligibility confirmation.

Baseline (T0)

- Anthropometrics and medication review.

- Full set of psychological questionnaires.
- Training in saliva and hair collection, diary instructions.
- Issuance of saliva collection kits and actigraphy devices (if used).
- Resting ECG/HRV and breathing measurement.

Between T0 and T1

- Eight weekly group sessions (INT or CTRL).
- Weekly collection of homework diaries and contact if sessions are missed.

T1 (~8 weeks)

- Repeat HRV/breathing assessments.
- Repeat psychological questionnaires.
- Three-day salivary sampling block.
- Hair sample (1-cm proximal segment).

T2 (~3 months from T0)

- Second three-day salivary sampling block.
- Psychological questionnaires and HRV (if feasible).

T3 (6 months from T0)

- Hair sample for HCC (1-cm proximal segment).

TSST substudy

In a subgroup:

- TSST is administered pre-intervention (pre-TSST) and post-intervention (post-TSST).
- Serial saliva samples are collected before, during, and after the stress tasks according to standard TSST timing.

10. Collection and handling of biological samples

10.1 Saliva

Sampling schedule per day

1. Immediately upon final awakening (0 min).

2. 30 min after awakening.
3. 45 min after awakening.
4. Daytime sample (~6–8 h after awakening).
5. Evening/bedtime sample (~30 min before sleep).

Compliance control

- Participants receive detailed written instructions with allowed time windows (±5–10 min for awakening samples; ±30 min for daytime/evening samples).
- Actigraphy and/or electronic time-stamps are used to verify awakening and sampling times.
- Restrictions: no food, caffeine, alcohol, smoking, or tooth-brushing during specified windows before sampling.

Laboratory processing

- Samples are frozen at −20 °C as soon as possible after return to the center, then stored at −80 °C until analysis.
- Analyses: salivary cortisol, cortisone, and α-amylase, using validated immunoassay/photometric methods.
- QC: LoD/LoQ, intra- and inter-assay CVs, plate/batch IDs, freeze–thaw cycles, time-to-freeze are documented.

10.2 Hair

- A 1-cm proximal hair segment is cut from the posterior vertex region (approximating 1 month of exposure).
- Samples are labeled and stored according to standard procedures.
- Cortisol is extracted and quantified; values reported as pg/mg.

11. Outcomes

11.1 Primary endpoint

Change in daily salivary cortisol AUCg (nmol/L·h), averaged across the three sampling days, from T0 to T1 (INT vs CTRL).

11.2 Key secondary physiological endpoints

1. Change in CAR AUCi from T0 to T1.

2. Change in diurnal cortisol slope (nmol/L per hour) from T0 to T1.
3. Change in salivary cortisone AUCg from T0 to T1.
4. Change in salivary α-amylase AUCg and slope from T0 to T1.
5. Changes in HCC (pg/mg) from T0 to T1 and from T0 to T3.

11.3 Mechanistic endpoints

1. Resting HRV indices (RMSSD, HFnu).
2. Resting breathing rate (breaths/min).
3. Questionnaire measures: ERQ-Reappraisal, Self-Compassion Scale (SCS), MAIA-2.

11.4 Clinical/psychological endpoints (secondary/exploratory)

1. BDI-II, STAI-Trait/State, perceived stress, and other self-report scales (as specified in the SOP).
2. Quality of life/functioning scales, if applicable.

11.5 TSST endpoints

- Peak salivary cortisol during TSST pre- and post-intervention and the change (post–pre) between groups.

11.6 Safety endpoints

- Frequency and nature of adverse events (AEs) and serious adverse events (SAEs).
- Negative effects of psychotherapy assessed by the Negative Effects Questionnaire (NEQ).

12. Sample size and power

Sample size calculations are based on the primary outcome: change in cortisol AUCg T0→T1.

Assumptions:

- minimally important between-group difference in change corresponds to effect size $d \approx 0.6$;
- two-sided $\alpha = 0.05$;
- power 80%;
- repeated-measures design and modest cluster effects (therapy group/therapist, $ICC \approx 0.03$).

Under these assumptions, approximately 25–30 participants per arm are required. Allowing for attrition, the target sample is **60 randomized participants** (INT n=30; CTRL n=30), with an upper bound of 80 if resources permit.

13. Statistical analysis plan (summary)

A detailed prespecified SAP is finalized before enrollment and attached as a separate document.

Key points:

1. **Analysis populations**
   - Primary analysis: intention-to-treat (ITT) including all randomized participants with available data.
   - Sensitivity analyses: per-protocol and complier average causal effect (CACE) analyses.
2. **Models**
   - Linear mixed-effects models (LMM) with fixed effects for time (T0, T1, T2), group, and time×group interaction.
   - Random intercepts for participant and therapy cluster (therapist nested within therapy group); random slopes for time as supported by model fit.
   - Hormonal outcomes analyzed on the log scale; diurnal slope on the raw scale.
3. **Covariates**
   - Awakening time, sampling-window adherence, caffeine/smoking indicators, plate/batch ID, freeze–thaw cycles.
   - For women, menstrual-cycle phase and OC use if needed.
4. **Multiple comparisons**
   - Benjamini–Hochberg false discovery rate (FDR, q=0.05) for prespecified secondary families (CAR/slope, sAA, cortisone, HCC, mechanistic targets).
5. **Missing data**
   - Mixed-model maximum likelihood under missing at random (MAR).
   - Multiple imputation and CACE analyses as robustness checks.
6. **TSST analyses**

   - Separate LMM/ANCOVA models for peak TSST cortisol with factors time (pre/post), group, and interaction.

7. **Software**

   - Analyses performed in R, Stata, SPSS, or equivalent statistical packages; code archived.

14. Data management and quality control

- Electronic case-report forms (eCRFs) capture awakening times (actigraphy), exact saliva sampling times, minutes-from-wake, window-compliance flags, protocol deviations, and contextual covariates (sleep, caffeine, smoking, alcohol, physical activity).
- Access to the de-identified analysis dataset is restricted to authorized study statisticians.
- Data quality checks include range checks, logic checks, and verification of extreme values.
- Laboratory QC: duplicate samples, control samples, calculation of intra- and inter-assay CVs, documentation of batch effects.

15. Safety monitoring

- All AEs and SAEs are recorded with onset date, severity, relationship to intervention, actions taken, and outcome.
- SAEs are reported to the Ethics Committee according to local regulations.
- The NEQ is used to systematically assess negative effects of psychotherapy.
- Participants with substantial clinical deterioration are referred to appropriate services; participation may be discontinued for safety reasons.

16. Ethical considerations

The trial is conducted in accordance with:

- the Declaration of Helsinki (latest revision);
- Good Clinical Practice (GCP);
- applicable national regulations of the Russian Federation.

The protocol, informed consent form, and related documents are approved by the Ethics Committee of the Center for New Medical Technologies (Decision No. 0182CS_2024 dated 10 December 2024).

Written informed consent is obtained from all participants before any study procedures. Personal data are kept confidential; only coded identifiers are used in analysis and publications.

17. Dissemination and data sharing

Study findings will be disseminated via peer-reviewed publications and scientific conferences.

De-identified individual participant data (IPD) and a codebook will be shared as supplementary material to the main publication; additional materials (e.g., analysis scripts) may be provided upon reasonable request and subject to data-use agreements and ethical approvals.
